# Artificial intelligence-driven multiple sclerosis diagnosis using genomic and medical imaging data: a review

**DOI:** 10.3389/fmed.2026.1812134

**Published:** 2026-10-29

**Authors:** Abdul Rahaman Wahab Sait, Yazeed Alkhurayyif

**Affiliations:** 1 Department of Documents and Archive, Center of Documents and Administrative Communication, King Faisal University, Al-Ahsa, Saudi Arabia; 2 Applied College, Shaqra University, Shaqra, Saudi Arabia

**Keywords:** artificial intelligence, deep learning, feature representation, gene expression, machine learning, medical imaging, multiple sclerosis diagnosis

## Abstract

Multiple Sclerosis (MS) is an immune-mediated central nervous system condition with heterogeneous clinical manifestations, progressive neurodegeneration, and considerable variability in disease trajectory. Recent advances in artificial intelligence (AI), particularly machine learning (ML) methods, have improved MS diagnosis and risk prediction. However, comprehensive review studies emphasizing the potential of existing AI-powered MS diagnosis remain limited. Thus, this study investigates the utilization of multimodal data by AI models to enhance diagnostic accuracy, evaluate disease progression, and optimize personalized care strategies. By adhering to PRISMA guidelines, this review included 26 studies encompassing MRI-based analysis, retinal OCT imaging, blood transcriptomics, SNP genotyping, and multimodal clinical datasets. An additional classification of these studies by modality was performed, and they were assessed with respect to dataset size, performance metrics, biomarkers, methodological robustness, and model interpretability. The findings indicate a consistent trend toward outstanding diagnostic performance, with some AI models achieving accuracies exceeding 95% using medical imaging and genomic data. However, limited external validation and interpretability procedures create a gap in clinical applicability and trustworthiness. This review highlights AI's growing potential to revolutionize MS diagnosis.

## 1 Introduction

Multiple sclerosis (MS) is a neurodegenerative and autoimmune disease that affects the central nervous system (1). It causes demyelination, axonal loss, and increasing neurological impairment as the disease progresses (2). This condition may manifest as a variety of clinical features (2). Secondary progressive MS and primary progressive MS are the most severe forms of the disorder. Despite its prevalence, MS is difficult to diagnose due to its varied symptomatology and comorbidity with other neurological conditions (3–5). To initiate disease-modifying therapies that can slow disease progression and improve patient outcomes, it is essential to make an accurate, early MS diagnosis (6). The inherent limitations of Conventional diagnostic methods, including laboratory biomarkers, magnetic resonance imaging (MRI), cerebrospinal fluid analysis, and clinical examination, reduce diagnostic accuracy (6).

Current MS diagnostic standards, including the McDonald Criteria, emphasize MRI to confirm lesion dissemination in space and time (7). However, interpreting these criteria is highly subjective, which may lead to misdiagnosis or delayed initiation of therapy (7). Furthermore, MRI is not an adequate predictor of disease progression or treatment response (7–10). Several genome-wide association studies (GWAS) have identified susceptibility loci, notably in the human leukocyte antigen complex (11–14). This suggests that genetic predisposition may contribute to MS etiology. Despite these advances, integrating genetic risk variables into a therapeutically meaningful prediction model remains an ongoing challenge (15–19).

Recent advances in understanding the disease pathology suggest that MS should be regarded as a continuum of pathological processes driven by complex interactions between neuroinflammation, neurodegeneration, and reparative mechanisms (21–23). The emerging paradigm has shown the limitations of classical diagnostic frameworks in terms of their ability to reflect the dynamics of changes in patients with MS. Artificial intelligence (AI) has emerged as a promising approach to overcoming existing limitations by integrating medical imaging, genetic, and clinical data, thereby transforming MS diagnosis (24–26). Machine learning (ML) algorithms have transformed multiple domains of healthcare, including neurology and neuroimaging (26). These techniques can analyze massive datasets, uncover complex patterns, and provide predictive insights, surpassing standard analytical methods. In MS diagnosis, AI-driven models have shown significant improvements in lesion segmentation, automated MRI interpretation, biomarker discovery, and predictive modeling of disease progression (1, 27–29).

Feature representation refers to converting raw biomedical data, such as images from magnetic resonance imaging (MRI) and optical coherence tomography (OCT) and genomic profiles, into quantitative features for analysis by ML algorithms (30–33). In terms of MS diagnosis, features could include lesion morphology, retinal layer thickness, and gene expression. Feature representation plays a critical role, as it determines an algorithm's efficacy, its ability to generalize, and its resistance to overfitting (34–38). Well-crafted representations improve an algorithm's performance and enhance its interpretability, allowing clinicians to understand the output in terms of clinically meaningful biomarkers (38).

Recent advances in AI-driven approaches have been applied to medical imaging, enabling automated lesion diagnosis with improved accuracy and repeatability. In contrast to time-consuming and inconsistent human lesion segmentation, AI-powered models enable high-throughput, consistent studies that enhance diagnostic precision (39–43). These models use longitudinal imaging data to predict the development of illness, allowing neurologists to tailor treatment plans (44). Advancements in neuroscience and biomarker studies have provided novel methods for quantifying MS-related disease activity (45–47). Quantitative and functional MRI techniques were shown to be more sensitive to microstructural and functional changes associated with disease progression (47). However, interpreting complex imaging signals in the context of disease progression remains a significant challenge for clinical practice.

In addition to imaging, AI models can simplify the integration of multi-omics data, including genetic, transcriptomic, and proteomic profiles, to provide a comprehensive understanding of MS patterns (47). Genomic and transcriptomic research has identified numerous markers for predicting patients' susceptibility to and progression of MS (48). However, there has been minimal success in interpreting these findings into therapeutic applications. By processing large-scale genetic databases and identifying novel biomarkers, AI-based algorithms can support precision medicine, offering personalized treatment based on individuals' genetic risk profiles. In addition, AI-powered natural language processing technologies may be used to explore EHRs and uncover subtle clinical trends, leading to earlier MS diagnosis (48).

Advances in machine learning architectures, such as transformer networks, have further enhanced neural networks' ability to model spatial and temporal dependencies in neuroimaging data. One of the key problems with current AI techniques is the lack of diverse, standardized, large-scale datasets needed to train models to predict MS. In addition, the increasing complexity of these models makes it difficult to analyze their decision-making processes and justify their decisions in high-stakes scenarios. The inherent clinical heterogeneity of MS poses a major issue in diagnostics and disease management since patients demonstrate highly divergent courses in terms of onset, progression, and responsiveness to treatment. Overall, these limitations require the development of appropriate methods for using AI models to analyze neuroimages of patients with MS, while ensuring high-quality, interpretable, and reliable results.

Although several systematic reviews have investigated the application of AI to the diagnosis of multiple sclerosis, most research has focused on either medical images or genetic data (48). There is a lack of comprehensive literature on how AI can enhance diagnosis accuracy by integrating genetics, imaging, and clinical data. In addition, the current research on the ethical concerns associated with MS diagnosis has a limited focus. These concerns include data privacy, model interpretability, and clinical acceptability.

Considering the fragmented nature of current reviews, a comprehensive, systematic review synthesizing findings across genetics- and medical imaging-based AI-driven MS diagnosis is essential. As data-sharing programs expand, there is a likelihood of developing multi-institutional artificial intelligence models that improve the generalizability and robustness of MS diagnostic approaches. These factors motivate us to evaluate recent advances in AI-driven MS diagnosis, highlight the limitations of current approaches, and propose future research to address knowledge gaps. This review introduces a comprehensive approach to MS diagnosis by integrating AI, genomics, and medical imaging into a unified framework. Using this distinctive approach, the study offers a broader, clinically relevant perspective on advances in precision medicine for MS. The study contributions are highlighted as follows:

1. Medical imaging and genomics biomarkers for early MS diagnosis.

This contribution promotes the development of AI-driven MS diagnosis models by identifying MS biomarkers. By enabling early intervention, long-term disability can be reduced, and an individual's quality of life can be improved.

2. Enhancing MS diagnosis model interpretability for clinical adoption.

This review focuses on techniques used in diagnostic systems, including feature attribution methods, decision visualization, and attention mechanisms. It highlights the biomarkers that influence the model's decision. The development of a transparent, traceable, and actionable MS diagnosis model can enhance clinician confidence, support informed decision-making, and address the gap between AI research and its implementation in clinical settings.

3. Emerging techniques for MS diagnosis.

This contribution highlights the significance of emerging techniques for improving model generalizability and robustness by providing access to larger, more varied datasets and by enhancing data security and compliance.

To ensure a consistent and thorough presentation of its objectives, techniques, and results, the remaining part of this systematic review is organized into four key sections. Section 2 details the search strategy, inclusion and exclusion criteria, data extraction process, and quality assessment techniques. During the selection and evaluation of studies, we follow PRISMA standards to ensure transparency, reproducibility, and scientific rigor. Section 3 provides an overview of the included studies, categorized by modality. It presents significant AI models, biomarkers, datasets, performance results, and study limitations. Within the context of the research topics, Section 4 discusses the possible clinical integration of the findings, the present challenges associated with deploying models, and the implications of AI-based MS diagnosis. Section 5 highlights the review's main findings, recommends the AI-integrated diagnostic paradigm, and suggests further studies.

## 2 Review methodology

By following the PRISMA guidelines, we evaluate the significance of AI-driven MS diagnosis based on genetics and medical imaging (49). The PRISMA guidelines provide a thorough framework for reporting and conducting systematic reviews, ensuring rigor in methodology, reliability, and transparency. These guidelines guarantee the study's robustness by outlining the processes for identifying, selecting, and evaluating relevant literature.

To ensure clarity, this study uses the terms AI, ML, and deep learning (DL) with specific distinctions throughout. AI refers to the broader field encompassing computational methods that enable systems to perform tasks requiring human-like intelligence. ML is a subset of AI that focuses on algorithms that learn patterns from data, such as support vector machines (SVMs) and random forests (RFs). DL is a further subset of ML that uses multi-layered neural networks, such as convolutional neural networks (CNNs), to automatically learn hierarchical feature representations from complex data. This distinction is maintained throughout the manuscript to avoid ambiguity and ensure methodological consistency.

### 2.1 Research questions

The treatment and diagnosis of MS entered an innovative phase due to advances in AI. There is an increasing demand for reliable, tailored, interpretable, and ethically acceptable diagnostic tools given the significant variation in clinical presentation, course, and response. By focusing on the following three research questions, this study intends to address its broader objectives.

Research Question (RQ1): How do AI models utilize medical imaging and genomic biomarkers for early MS diagnosis?
Research Question (RQ2): Can AI models improve the interpretability and clinical trustworthiness of MS diagnosis?
Research Question (RQ3): What are the emerging techniques to enhance MS diagnosis while preserving data privacy and improving generalizability?

### 2.2 Inclusion and exclusion criteria

To ensure the relevance of the selected studies, we developed inclusion and exclusion criteria. Table 1 outlines the inclusion and exclusion criteria, emphasizing AI-enabled feature representation and classification for MS diagnosis.

**Table 1:** Inclusion and exclusion criteria for study selection.

Criterion	Inclusion	Exclusion
Study Type	Peer-reviewed research articles.	Review and Non-peer-reviewed articles.
Language	Published in English.	Non-English studies.
Focus	Emphasizes the use of genomic data, medical imaging, and multimodality with AI in MS diagnosis.	Exclusively investigates the genome or imaging without AI.
Methodology	Clearly defined methodology with a sufficient dataset for reproducibility.	Limited methodological details for evaluation
Sample Size	Studies with ≥30 participants for statistical robustness.	Studies with < 30 participants.
Data Availability	Studies providing sufficient data for interpretation.	Conference abstracts without full-text availability.

Summary of predefined criteria used to include or exclude studies in this review. Inclusion criteria required studies employing AI-based models for MS diagnosis or prediction using imaging, genomic, or clinical data, with sufficient methodological transparency and sample size (≥30 participants). Exclusion criteria included reviews, studies lacking validation, insufficient reporting, or non-clinical relevance. Key observation: Criteria ensure methodological rigor and reproducibility.

### 2.3 Search strategy

The author systematically searched academic databases such as PubMed, Scopus, Web of Science, and IEEE Xplore to identify relevant studies. The extensive coverage of medical, genetic, and AI-related research in these databases is a key factor in their selection. Additional searches in the Google Scholar database help ensure comprehensive literature retrieval. A thorough examination of the references in the selected studies was conducted to identify additional relevant studies. The search strategy was adapted to the indexing structure of each database, with truncation and wildcard operators applied where appropriate to maximize retrieval sensitivity. The final database search was conducted on 12.10.2025. To ensure adequate representation of the two principal domains considered in this review, complementary modality-specific searches were also performed.

Medical Subject Headings terminology and pertinent keywords guided an organized search approach. For this search, the following Boolean operators and search terms were utilized:

(“multisclerosis” OR “MS”) AND (“genetics” OR “genomics” OR “genetic markers”) AND (“medical imaging” OR “MRI” OR “radiology”) AND (“artificial intelligence” OR “machine learning” OR “deep learning” OR “AI”).

The author tailored the search approach according to each database's indexing system. In order to achieve the highest possible level of search sensitivity, truncation and wildcards were applied.

### 2.4 Study selection

The study selection method included title and abstract screening, followed by full-text evaluation. Using the eligibility criteria, two independent reviewers examined the search output. When there were disagreements about selection judgments, they were resolved through conversation or consultation with a third reviewer. To provide a visual depiction of the screening process, a PRISMA flow diagram was used to record the inclusion of studies. This diagram shows the number of records identified, screened, excluded, and included in the final review.

### 2.5 Data collection process

A standardized data extraction form was developed to ensure uniformity in data collection. Data, including authors, publication year, research design, sample size, AI techniques, genetic markers investigated, and imaging modalities, were utilized, and MS diagnostic results were retrieved. To simplify organization and tracking, the authors assigned a unique identifier to each study.

### 2.6 Risk of bias and quality assessment

To achieve methodological rigor, the quality of the selected literature was assessed through systematic critical appraisal using the QUADAS-2 (Quality Assessment of Diagnostic Accuracy Studies) criteria. QUADAS-2 is a highly validated tool for systematically appraising diagnostic studies, including those involving AI models. QUADAS-2 includes four key areas where potential risks of bias were identified: patient selection, index test (implementation of AI models), reference standard (ground truth), and flow and timing. Each study was independently assessed and classified as having low, high, or unclear risk of bias within each domain. Particular attention was given to dataset representativeness, validation strategies, and the reproducibility of AI pipelines. The detailed results of this assessment are presented in Supplementary Table S1, which provides a comprehensive and transparent evaluation of methodological quality across all included studies.

### 2.7 Semi-quantitative methodological evaluation framework

To overcome heterogeneity and enable methodological comparison across studies, a semi-quantitative assessment approach was adopted. In addition to the basic description, the methodological approach allows systematic evaluation of the robustness and clinical feasibility of the individual studies. The individual studies were assessed regarding dataset size, validation approach, interpretability, data diversity, and modality integration. For dataset size, classification into small (<100 patients), medium (100–500 patients), and large (>500 patients) was used. Furthermore, studies were rated by validation strategy, whether internal (e.g., cross-validation) or external (independent cohort validation). The use of interpretability techniques, such as SHAP, LIME, and other feature-attribution methods, was considered. Moreover, data diversity was assessed for single-center vs. multi-center datasets, and modality integration for single-modal vs. multimodal approaches. Based on the above criteria, an overall methodological score of 0–5 was assigned to each study.

### 2.8 Comparative synthesis and study ranking

The methodological characteristics of the studies, including dataset size, types of validation, external validation, interpretability methods, and risk of bias, were assessed systematically using the risk-of-bias criteria from Supplementary Table S1 and the criteria set out in Supplementary Supplementary Table S2. Based on the methodology scores, the studies were grouped into three rigor groups. Studies with scores ≥4 were considered high in rigor, indicating strong methodology and potential for clinical use. With scores ranging from 2 to 3, the studies were considered to have moderate methodology with limited generalizability, while those with scores ≤1 were regarded as having low rigor, implying a higher risk of bias.

## 3 Results

To demonstrate the methodological transparency and integrity of the review process, the PRISMA flowchart is crucial. From initial search to final inclusion, Figure 1 describes each stage of study selection in this systematic review examining the use of AI in MS diagnosis using genetic and medical imaging data.

**Figure 1:**
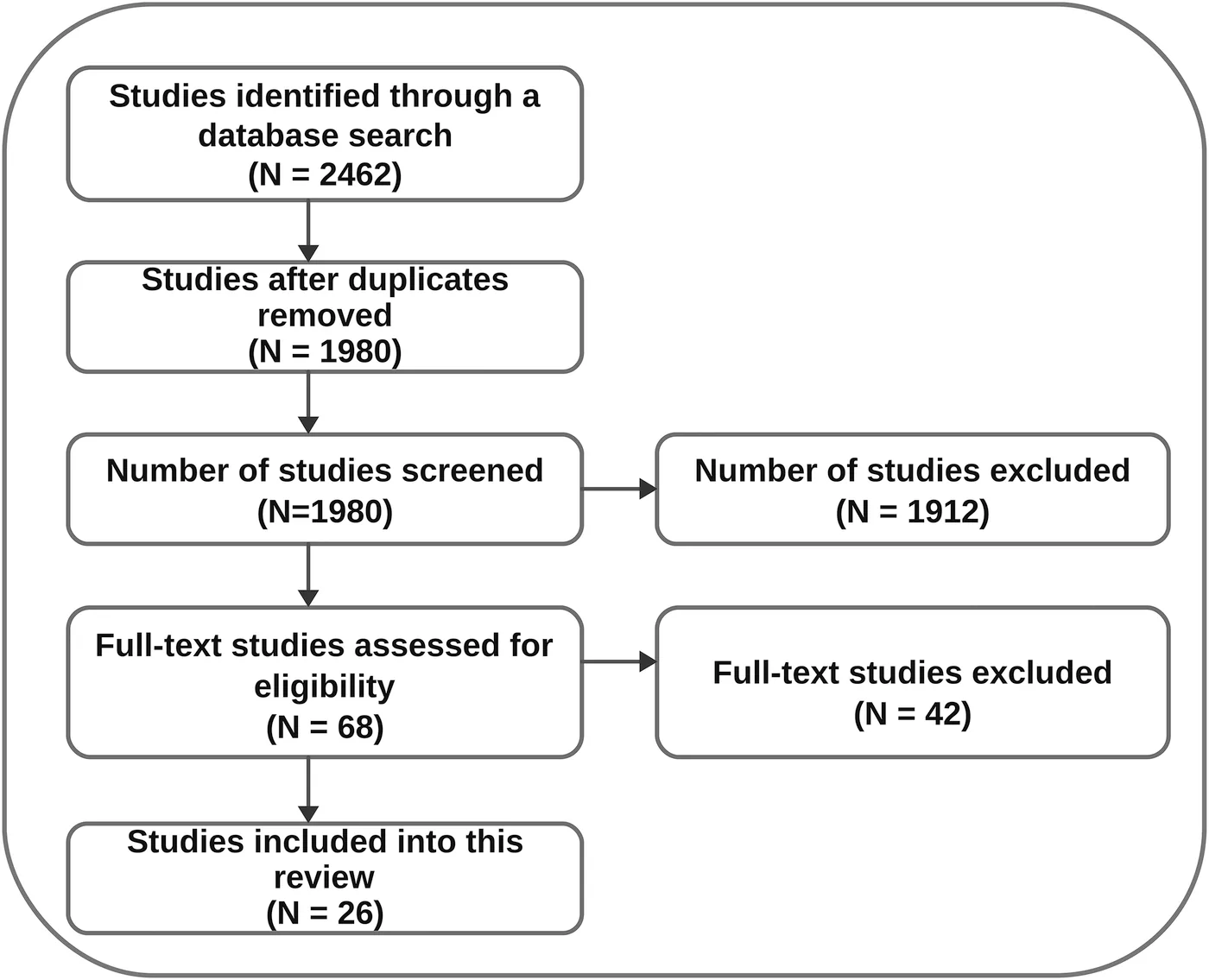
PRISMA-based study selection workflow for AI-based MS diagnosis. Flow diagram illustrating the study selection process. A total of 2,462 records were identified through searching process, reduced to 1,980 after duplicate removal. Following title/abstract screening, 1,912 studies were excluded, and 68 full-text articles were assessed for eligibility. Of these, 42 were excluded based on predefined criteria, resulting in 26 included studies. Arrows indicate sequential filtering stages. Key observation: The substantial reduction highlights strict inclusion criteria emphasizing methodological rigor and reproducibility.

Multiple reputable databases, including PubMed (*n* = 1,022), Scopus (*n* = 876), Web of Science (*n* = 354), and IEEE Xplore (*n* = 210), were searched to conduct a comprehensive literature review. A multidisciplinary approach was ensured by selecting these resources, which encompass viewpoints on MS diagnosis from the clinical, genetic, computational, and engineering domains. The total number of records returned by the initial search was 2,462. Following the removal of 482 duplicates, 1,980 unique studies underwent thorough screening of titles and abstracts. Following the initial screening, 1,912 publications were disqualified for lacking AI integration, not emphasizing MS, or lacking computational methodologies for imaging or genetics. Subsequently, 68 full-text publications were evaluated to determine eligibility. Out of these, 42 were deemed ineligible for a variety of reasons, including the absence of full-text availability, inadequate methodological clarity, the absence of peer-reviewed sources, or the inability to fulfill the multimodal or AI criteria. A total of 26 high-quality studies were ultimately included in the final synthesis. Using AI or machine learning to diagnose or predict MS using MRI, OCT, retinal imaging, or genetic data, these studies satisfied all inclusion requirements. By providing a framework for this complex filtering process, the PRISMA flowchart ensures readers that the literature was selected in a methodical, impartial, and evidence-based manner. Through this methodological rigor, the review's contributions to advancing AI-powered precision diagnostics for multiple sclerosis are strengthened.

Although the preliminary search returned 2,462 records, only 26 studies met the full-text inclusion criteria. This stringent selection process is consistent with a quality-focused strategy that demands the inclusion of studies that use artificial intelligence in the development of diagnostic or predictive models, incorporate imaging or genomic information, have an adequate sample size (minimum of 30 subjects), and provide sufficient methodological clarity to ensure replicability. Many studies were not included because they lacked detailed methodologies, validation, or clinically relevant metrics.

Figure 2 demonstrates a positive trend in publication volume, underscoring the review's timeliness and relevance. As the healthcare industry moves toward AI-integrated clinical processes, this finding indicates an increasing number of studies using computational tools to develop personalized, explainable diagnostic systems.

**Figure 2:**
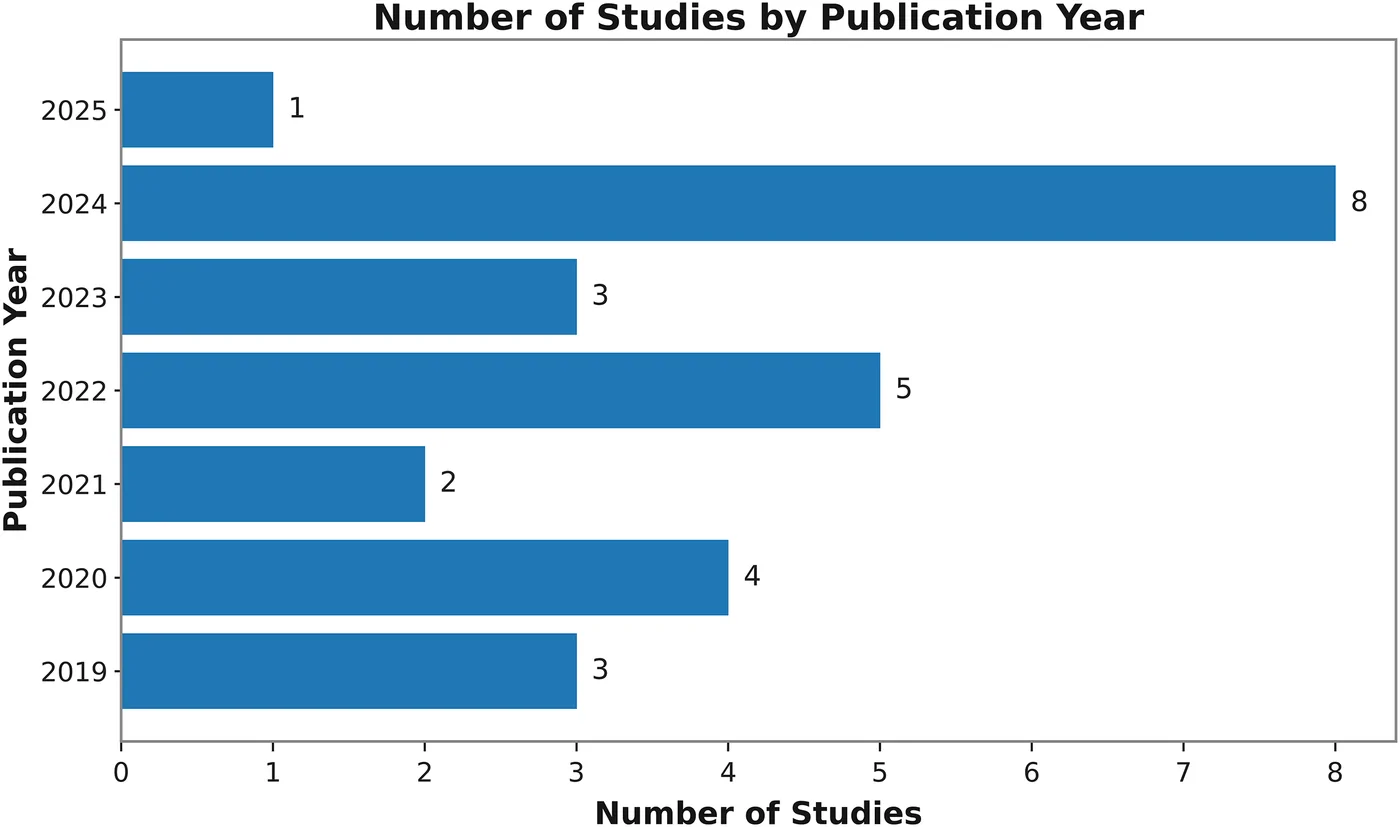
Year-wise distribution of included studies (2019–2025). Horizontal bar chart showing the number of included studies per publication year. The *x*-axis represents the number of studies, and the *y*-axis represents the publication year. A notable increase is observed in 2024 (*n* = 8), followed by a gradual increase in earlier years. Key observation: The trend reflects a rapid increase in research interest in AI-driven MS diagnosis over recent years.

Figure 3 offers a graphical representation of the included studies. These studies were categorized by the primary data modality used in AI-based MS diagnosis. It highlights the progression of AI applications in MS, showing a distinct trend toward genetic data and retinal imaging as novel avenues of MS diagnosis. In addition, it sheds light on relatively unexplored topics such as MRI-AI fusion and cross-modal systems, paving the way for future multimodal research. The wide range of biological data types used to explore diagnostic improvements in MS research is reflected in the four classifications: MRI, genomic data, OCT, and others. Among these groups, OCT is the most prominent modality with 13 studies. Retinal imaging is increasingly relied upon as a non-invasive, highly sensitive tool for detecting neurodegenerative changes associated with MS. In clinical settings, OCT is a viable, scalable solution because of its cost-effectiveness, accessibility, and direct association with neuroaxonal loss. Over the last several years, OCT adoption has increased as AI models, particularly deep learning, have been integrated with OCT data. This integration additionally enhances the diagnostic accuracy of OCT. Genomic data from six studies reveal an additional frontier in MS research. To analyze high-dimensional genomic and transcriptomic information, these studies use ML techniques. This enables prediction of disease onset, progression, and treatment response. Genomic data support the growing trend toward precision medicine, characterized by the use of an individual's molecular profile in diagnostic decision-making. Only three MRI-based studies are included, despite their importance in MS diagnosis. The challenges of using AI in MRI settings include data complexity, acquisition costs, and interoperability across centers. The “Others” category contains four studies on multimodal integrations and clinical data.

**Figure 3:**
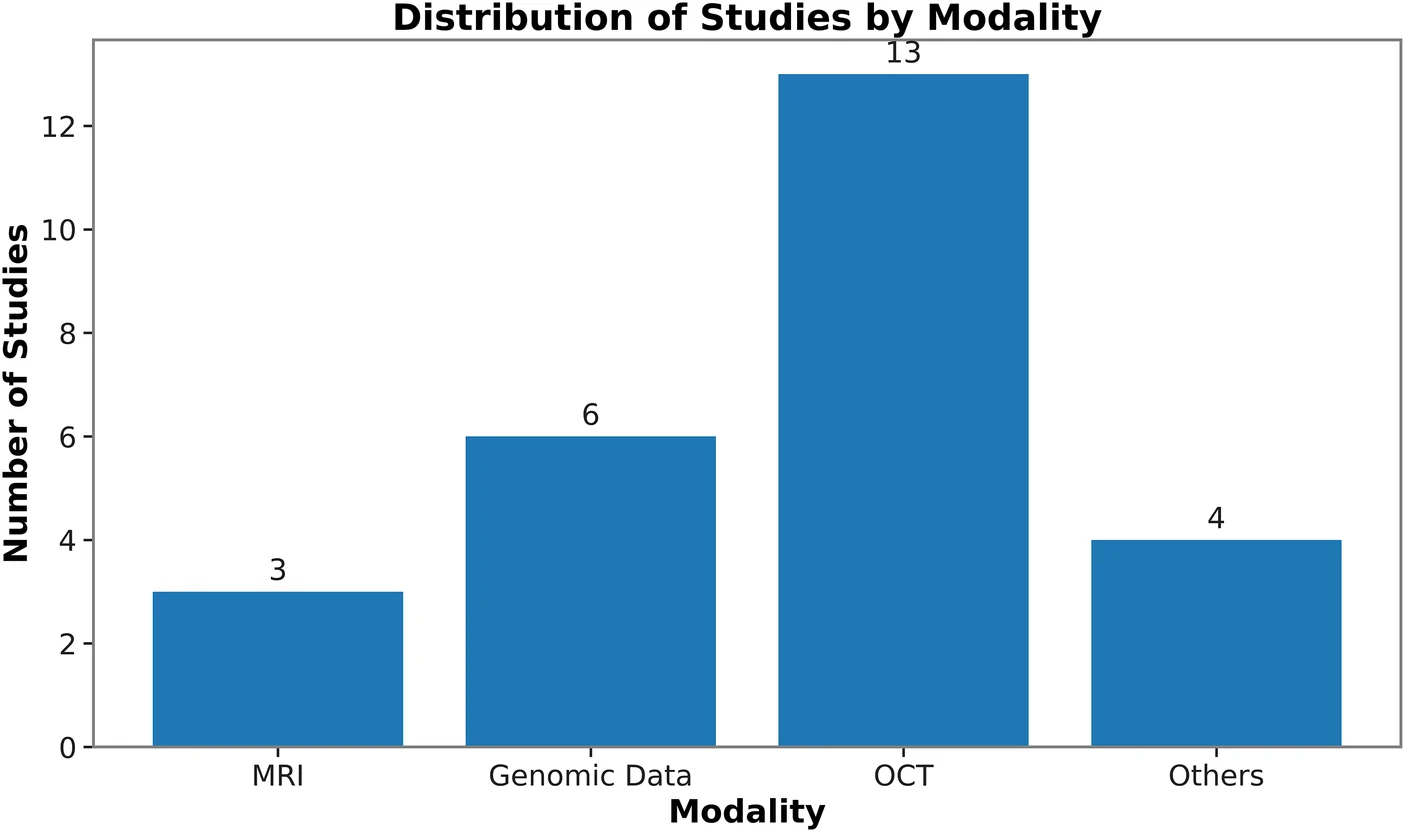
Distribution of studies across diagnostic modalities. Bar chart illustrating the number of studies categorized by modality: MRI, genomic data, OCT, and other modalities. The *x*-axis denotes modality type, and the *y*-axis indicates study count. Key observation: OCT and genomic approaches are more represented, indicating a shift toward non-invasive, data-rich diagnostic strategies.

### 3.1 RQ1: performance of AI models across modalities

#### 3.1.1 MRI-based MS diagnosis

Table 2 provides insight into the current state of MRI-based artificial intelligence models for diagnosing MS patients, offering additional information on the mechanics of model construction and testing. A significant observation is that deep learning architectures, such as ResNet-50 (50), exhibit strong feature-extraction capabilities using MRI images. In particular, these algorithms excel in detecting atrophy and lesions in gray matter. These capabilities explain their relatively high efficiency compared with classical machine learning models that use handcrafted radiomic features (51).

**Table 2:** MRI-based AI models for MS diagnosis: methods, performance, and limitations.

Study	Model	Performance	Key biomarkers	Limitations	Dataset
Huang et al. (50)	RF, 10-fold cross-validation	AUC (Area Under Curve): 0.90; Accuracy: 0.87; Sensitivity: 0.87; Specificity: 0.87	T1/T2 radiomic features + clinical data	Small dataset; no external validation; MRI variability	*n* = 116 (78 MS, 38 Neuromyelitis Optica)
Taloni et al. (51)	ResNet-50 (Machine Learning, 3D T1-weighted MRI)	AUC: 0.81 (Sagittal), 0.72 (Coronal), 0.69 (Axial)	Gray matter atrophy, frontal regions, Expanded Disability Status Scale (EDSS)	Limited generalization; single modality; EDSS bias	*n* = 181 (2 centers, Italy)
Gaviraghi et al. (52)	U-Net (Machine Learning for diffusion-weighted MRI)	Root Mean Square Error (RMSE): 0.046 vs. 0.325; Structural Similarity Index (SSIM): 0.85 vs. 0.24	Fractional Anisotropy (FA), white matter integrity	Limited generalizability; preprocessing and resolution dependence	*n* = 123 (UK)

Comparison of studies utilizing MRI data, including model type (e.g., RF, CNNs, U-Net), performance metrics [e.g., area under the curve (AUC), accuracy], key imaging biomarkers, limitations, and dataset characteristics. Key observation: High performance is often constrained by small datasets and a lack of external validation.

However, these models' performance is typically hindered by limited dataset size and the lack of external validation, thus increasing the risk of overfitting. By contrast, radiomics models are characterized by higher interpretability, as they can detect clinically significant imaging biomarkers, though they are less effective at finding patterns in complex structures. Another class of successful algorithms is U-Net-based models, which use an encoder-decoder architecture and perform effectively for reconstruction and segmentation. However, their high sensitivity to image processing techniques is a potential problem.

A critical trade-off emerges between predictive performance and interpretability, where highly accurate deep learning models often lack transparency, limiting clinical trust and adoption. Furthermore, systematic limitations across studies—including homogeneous cohorts, variability in MRI acquisition protocols, and reliance on internal validation—restrict the translation of these models into real-world clinical settings. Collectively, these findings highlight that while MRI-based AI models achieve high diagnostic performance, their clinical applicability is constrained by methodological and validation challenges, not by algorithmic capability alone.

ML algorithms show high accuracy, achieving mean AUC scores of 0.70–0.90 across the included studies. The major biomarkers used include lesion volume, location, and gray matter atrophy, which are associated with the severity of MS symptoms and disease progression. The studies use small, single-center datasets and validate using internal methods such as cross-validation, which may limit generalizability. Differences in MRI acquisition procedures and pre-processing steps could add bias. Although AI models improve feature extraction, the resulting features can be difficult to interpret, thereby limiting their usefulness in clinical practice. Additionally, variability in MRI imaging protocols and preprocessing steps may introduce biases. Despite improved performance and feature extraction by DL models, the lack of interpretability can negatively affect clinical trust.

#### 3.1.2 Genome-based MS diagnosis

Strategies leveraging AI techniques are effective solutions for addressing inherent problems in genomic data associated with multiple sclerosis, primarily high dimensionality and polygenicity—numerous genetic variants involved in the development of this condition, each affecting it in a minor, correlated way. Standard statistical algorithms often fail in situations with an imbalanced feature space and a limited sample size. In machine learning models, including Random Forests and gradient boosting, feature selection enables biomarker discovery and helps suppress noise. For instance, Acquaviva et al. (53) demonstrated high sensitivity (94.3%). However, this result is influenced by the lack of external validation and by overfitting issues associated with small dataset sizes. Similarly, Omrani et al. (54) achieved impressive results (classification accuracy of 97%) using transcriptomic and phenotypic features. Still, batch effects and dataset heterogeneity are important issues limiting the reproducibility of this research.

From a mechanistic point of view, models using genomics significantly exceed the ability of simple models to analyze non-linear gene-to-gene and gene-to-environment interactions crucial for MS development. Another limitation is the low biological interpretability of the biomarkers identified. An illustrative example is the study by Fuh-Ngwa et al. (55), which reported high accuracy (*r* = 0.86) but left the exact role of SNPs unknown.

A consistent failure mode is the risk of overfitting due to high dimensionality, compounded by small sample sizes and population-specific datasets (56), which restricts generalizability across diverse cohorts. Additionally, a trade-off exists between model complexity and interpretability: advanced models improve predictive performance but offer limited biological insight, hindering clinical translation (57, 58). Multimodal integration partially addresses these challenges by combining genomic, transcriptomic, and clinical data, enabling more comprehensive modeling of disease mechanisms. However, this introduces additional complexity, including data harmonization issues and increased susceptibility to bias. Overall, while genomics-driven AI models demonstrate strong potential, their clinical applicability remains constrained by validation gaps, interpretability limitations, and underlying biological uncertainty rather than purely predictive performance.

Although most ML models exhibit high accuracy, this should be taken with caution, given the inherent limitations within the methods used. A notable limitation is the imbalance between feature dimensionality and sample size across the studies, which increases the risk of overfitting. Other limitations include the lack of validation across independent datasets and populations. Despite these problems, genomic models can provide important biological insights, including the identification of immunological pathways and risk genes. Table 3 offers the Genomics-based AI models for MS diagnosis and biomarker discovery.

**Table 3:** Genomics-based AI models for MS diagnosis and biomarker discovery.

Study	Technology	Performance	Key biomarkers	Limitations	Dataset
Acquaviva et al. (53)	Ensemble ML (Functional Trees, RF, AdaBoost) with Nested Cross-Validation (NCV)	Sensitivity: 94.3%; Precision: 87.5%	Transcriptomic signatures (139 features); 213 gene probes (CIS); 222 markers (Progressive MS)	Overfitting risk; no external validation	*n* = 313 (blood transcriptome)
Omrani et al. (54)	ML (AdaBoost, RF) + glmboost feature selection	Accuracy: 97%; C-index: 0.74 (CIS prediction)	107 transcriptomic signatures; 58 CIS predictors; lncRNA, miRNA, snoRNA	Limited generalizability; batch effects	*n* = 225 (RNA-seq)
Fuh-Ngwa et al. (55)	Ensemble ML + Mixed-Effect ML (MEML)	Correlation: *r* = 0.86 (*p* < 0.001) for disability prediction	7 SNPs loci linked to MS progression	Unclear biological significance; overfitting risk	*n* = 279 (Australian cohort)
Ghafouri-Fard et al. (56)	Artificial Neural Network (ANN) with 5-fold CV + LIME (interpretability)	Accuracy: 64.7%; Sensitivity: 65.0%; Specificity: 64.5%; AUC: 0.70	Risk SNPs: ACE, CBLB, VEGFA; Protective SNPs: GAS5, MALAT1	Limited SNP set; population bias (Iranian cohort)	*n* = 791 (401 MS, 390 controls)
Jeyananthan (57)	ML + Mutual Information feature selection	Accuracy: 0.88 (phenotype); 0.94 (therapy); RMSE: 4.1	Gene expression patterns for MS subtypes and therapy response	No external validation; small dataset	*n* = 78 (transcriptome)
Arnal Segura et al. (58)	Explainable ML/DL + Recursive Feature Elimination	Balanced accuracy: 0.60–0.70	Immune-related genes: HLA-A02:01, HLA-DRB115:01, IL7R, CD226	Performance variability; no external validation	UK Biobank (genotyping data)

Overview of studies employing genomic and transcriptomic data, highlighting machine learning techniques, performance outcomes, identified biomarkers, and methodological limitations. Key observation: While models effectively handle high-dimensional data, generalizability and biological interpretability remain key challenges.

#### 3.1.3 OCT-based MS diagnosis

Table 4 summarizes recent advancements in AI-assisted MS diagnosis using OCT features, elucidating important methodological and clinical insights beyond performance metrics alone. Unlike MRI and genomics, OCT provides highly consistent, low-dimensional, and anatomically local data (20, 59–70). It explains why AI models tend to perform exceptionally well, regularly achieving accuracies higher than 85%. CNNs excel at recognizing structural differences in the OCT images of retinal layers. They include the retinal nerve fiber layer (RNFL) thinning and Ganglion cell and inner plexiform layer (GCIPL) thickness, which are directly related to neuroaxonal degeneration; hence, the high stability and performance of OCT-based models compared to complex imaging modalities.

**Table 4:** OCT-based AI models for non-invasive MS diagnosis.

Study	Technology	Performance	Key biomarkers	Limitations	Dataset
Montolío et al. (59)	ML + LSTM + Ensemble Classifier (EC)	Accuracy: 87.7%; Sensitivity: 87.0%; Specificity: 88.5%; AUC: 0.88	RNFL thickness (quadrants), EDSS	Small, single-center dataset	*n* = 212
Pérez del Palomar et al. (60)	SS-OCT (Swept-Source OCT)	Accuracy: 97.2%; AUC: 0.995	Macular RNFL thickness	No longitudinal analysis	*n* = 260
He et al. (61)	Deep Learning (S-Net + R-Net)	Dice score: 0.64 (MME segmentation)	Retinal layers (RNFL, GCL, INL, etc.)	Limited data; device variability	*n* = 35
Khodabandeh et al.(62)	CNN + Autoencoder + Transfer Learning (ResNet152V2)	Accuracy: 97.3%; Specificity: 97.3%; Recall: 97.4%	RNFL, GCIPL, INL	Acquisition variability; dataset bias	*n* = 116
Ciftci Kavaklioglu et al. (63)	RF + Feature Selection	Accuracy: 80% (MS vs. HC)	RNFL (temporal), GCIPL, macular features	Single-center; disease variability	*n* = 699
Ortiz et al. (64)	CNN	Accuracy: 87%; Sensitivity: 82%; Specificity: 92%	GCL thickness, IPL asymmetry	Limited cohort; no progressive MS	*n* = 148 (RRMS + HC)
Arian et al. (65)	CNN + Autoencoder (CAE + MLP)	Accuracy: 88%; AUROC: 0.94	RNFL, GCIPL thinning	Small dataset; no longitudinal data	Multi-dataset (∼349 images)
Aghababaei et al. (66)	CNN (VGG, ResNet, Inception)	Accuracy: 85%; Sensitivity: 85%; Specificity: 87%	RNFL, GCIPL, vessel density	No ON differentiation; no multimodal data	*n* = 314
Montolío et al. (67)	CNN + LASSO feature selection	Diagnosis: Acc: 90.3%; Prognosis: Acc: 81.9%	Macular GCL regions, EDSS	Small, homogeneous cohort	*n* = 102 participants
Dongil-Moreno et al. (68)	SVM + Recursive Feature Elimination (RFE)	Sensitivity: 86%; Specificity: 90%	Retinal asymmetry, papillomacular bundle	No longitudinal data	*n* = 148
Kenney et al. (20)	SVM + Logistic Regression	AUC: 0.89; Sensitivity: 81%; Specificity: 80%	GCIPL thickness, inter-eye difference	Moderate sensitivity; cross-sectional	*n* = 2,120 (multi-site)
Cavaliere et al. (69)	SVM	Accuracy: 91%; AUC: 0.97	GCL thickness, macular retina	Small dataset; no MRI correlation	*n* = 96
Yaghoubi et al. (70)	MobileNetV2	DL Accuracy: 98%; AUC: 0.96	Retinal vessel texture features	Small dataset; image dependency	*n* = 265

Summary of studies using OCT, including model architectures, performance metrics, retinal biomarkers, and dataset characteristics. Key observation: Standardized retinal features enable high diagnostic accuracy but are limited in their ability to capture systemic disease heterogeneity.

There are several limitations of OCT-based models despite their high diagnostic accuracy. First, the narrow focus on biomarkers limits generalizability to broader patient populations, as many studies report excellent performance but low sensitivity to other MS-related pathologies. In addition, single-center studies often employ cross-sectional designs, which limit the predictive power of OCT models and introduce variability in acquisition devices and segmentation methods. There is an intrinsic trade-off between the interpretability of input features and predictive power. On the one hand, OCT features (unlike imaging and genomic features) are biologically interpretable; on the other hand, this interpretability may result in suboptimal model flexibility needed for accurate prognosis. Least absolute shrinkage and selection operator (LASSO) and recursive feature elimination may help improve model performance at the cost of excluding important, yet weakly represented variables.

Overall, OCT-based AI models demonstrate strong potential for scalable, non-invasive screening, yet their clinical utility is best realized when integrated with complementary modalities rather than as standalone diagnostic systems.

Compared with MRI, OCT results tend to show higher classification accuracy, which may be attributable to a consistent data-collection protocol and the dataset's reduced dimensionality. Commonly used biomarkers are RNFL thickness and ganglion cell layer thickness, both of which provide sensitive measures of axonal damage. Many researchers use machine learning algorithms trained on moderately to large-sized datasets, while some validate their methods through a multi-center approach. However, OCT techniques are relatively limited in their ability to detect central nervous system pathology extending beyond the visual system. Interpretability is also relatively weak, with few investigations providing insights regarding feature importance. OCT provides an affordable, noninvasive solution for disease diagnosis but may not be sufficient alone, necessitating multimodal MS diagnosis.

#### 3.1.4 Other modalities-based MS diagnosis

Table 5 describes the contributions of AI-based models to integrating clinical, functional, and biological data for diagnosing and predicting MS. Unlike single-modality models, AI-based models can incorporate data from multiple modalities that complement each other from both phenotypic and biological perspectives. For example, the inclusion of imaging, retinal, and cytokine data significantly increases the model's accuracy (Andorra et al. (71); AUC up to 0.85). Furthermore, there are cases when only clinical data are used to improve the model's performance [Pinto et al. (72); Pellegrini et al. (73)]. The studies demonstrate that longitudinal measures, such as the EDSS, are important for building models that forecast disease progression.

**Table 5:** Multimodal and clinical AI models for MS diagnosis and prognosis.

Study	Technology	Performance	Key biomarkers	Limitations	Dataset
Andorra et al. (71)	Multimodal RF	AUC: 0.80 (NEDA), 0.77 (EDSS), 0.85 (SLCT)	MRI brain volume, RNFL (Retinal Nerve Fiber Layer), cytokines	Missing key biomarkers (NfL, OCB); no spinal MRI; limited sample	*n* = 420
Pinto et al. (72)	ML (SVM, Decision Tree, k-NN, Linear Regression)	AUC: 0.86 (2 years), 0.89 (6 years), 0.85 (10 years)	EDSS, functional scores, age at onset	Missing data; no MRI/genomics; limited diversity	*n* = 187 (clinical cohort)
Pellegrini et al. (73)	Statistical + ML (LASSO, Ridge, Elastic Net, SVM-Cox, RF)	C-index ≤ 0.65	PASAT, SF-36 PCS, VFT, MSFC	Low predictive power; inconsistent features; no external validation	*n* = 1,582
Ferrè et al. (74)	Multiview RF (Genetic + Clinical)	AUROC: 0.71 (combined); Accuracy: 66%	SNPs (immune/inflammatory genes), clinical features (ARR, lesions)	Moderate performance; high dimensionality; small cohort	*n* = 381 (multi-center)

Comparison of studies integrating clinical, imaging, and biological data, including model types, predictive performance, and limitations. Key observation: Multimodal integration improves personalization and predictive capability but introduces challenges related to data heterogeneity and missing modalities.

However, these approaches also reveal key limitations. Models relying solely on clinical data often exhibit moderate predictive performance, as they lack the biological specificity provided by imaging or genomic features. This is evident in the relatively low discrimination power reported by Pellegrini et al. (73), suggesting that clinical variables alone may be insufficient for robust diagnosis. Conversely, multimodal models, such as Ferrè et al. (74), improve performance by integrating genetic and clinical data, but introduce challenges related to data heterogeneity, missing modalities, and increased model complexity.

A critical insight is that multimodal integration improves generalizability by capturing diverse disease mechanisms. However, it amplifies sources of bias and variability, particularly when datasets are incomplete or unbalanced. Additionally, a trade-off arises between model complexity and clinical interpretability: highly integrated models may achieve better performance but become less transparent and harder to implement in routine practice.

Multimodal AI techniques represent an incredibly promising trend in MS diagnostics, thanks to their ability to exploit complementary information from imaging, genomics, and clinical data sources. Empirical findings from multimodal models integrating MRI, OCT, and molecular data indicate that predictive performance is superior to that of unimodal approaches. Superiority is achieved through the ability to detect both structural and biology-related aspects of disease pathology. The reason for this improvement lies in the ability to use different perspectives on disease pathogenesis. Moreover, the use of multiple data sources makes such models more robust to missing and noisy data and increases their generalizability when validated across diverse samples. However, these methods require more sophisticated data processing and come with the problem of increased model complexity. Thus, they become harder to interpret and apply clinically. Still, multimodal AI approaches resonate with the concept of precision medicine.

### 3.2 RQ2: methodological characteristics and limitations

In answering RQ2, the impact of the methods used on the effectiveness of AI models for MS diagnosis using biomarkers should be evaluated. The literature review suggests that a wide range of biomarkers can serve as important variables for AI models in MS diagnostics. These biomarkers include MRI features related to lesion characteristics, OCT-based metrics of retinal layer thickness, and genomic information on gene expression profiles. These biomarkers offer complementary insights regarding the structural, functional, and molecular aspects of the disease pathology, enabling predictive modeling. Based on the biomarker representations, different AI methods demonstrate varied capabilities in extracting and using the required features.

DL models such as CNNs achieve strong performance on imaging-related tasks because they can automatically learn spatial hierarchies, as observed in MRI studies. Conversely, classical machine learning algorithms such as SVMs and RFs are commonly used in structured or high-dimensional settings, such as genomics and radiomics-derived MRI analyses, where feature engineering is crucial. Moreover, ensemble models further improve reliability by combining multiple models, particularly when dealing with multimodal information. However, numerous methodological issues still exist in most of the reviewed papers. Most of them use small, single-center datasets alongside internal validation strategies, increasing the risk of overfitting while limiting generalizability. Moreover, variability in pre-processing could lead to data leakage, especially when analyzing images. Finally, the lack of interpretability in many deep learning models impedes their clinical use because their predictions cannot be directly linked to biological mechanisms.

#### 3.2.1 MRI biomarkers

MRI-based biomarkers play a crucial role in the diagnostic pathway for multiple sclerosis, providing quantifiable, localized insight into pathogenesis. FA acts as an index of white matter integrity and helps assess microstructural lesions. The volume of brain lesions and their localization serve as the key markers of disease and have proven correlation with disability progression. Gray matter atrophy in cortical and thalamic regions is an early marker of neurodegeneration and cognitive impairment. Multi-parametric features from MRI analysis, derived from combinations of T1, T2, and FLAIR sequences, improve diagnostic and prognostic precision. Overall, MRI biomarkers contribute to developing an evaluation system that ensures timely diagnosis, treatment planning, and disease monitoring in MS patients (Figure 4).

**Figure 4:**
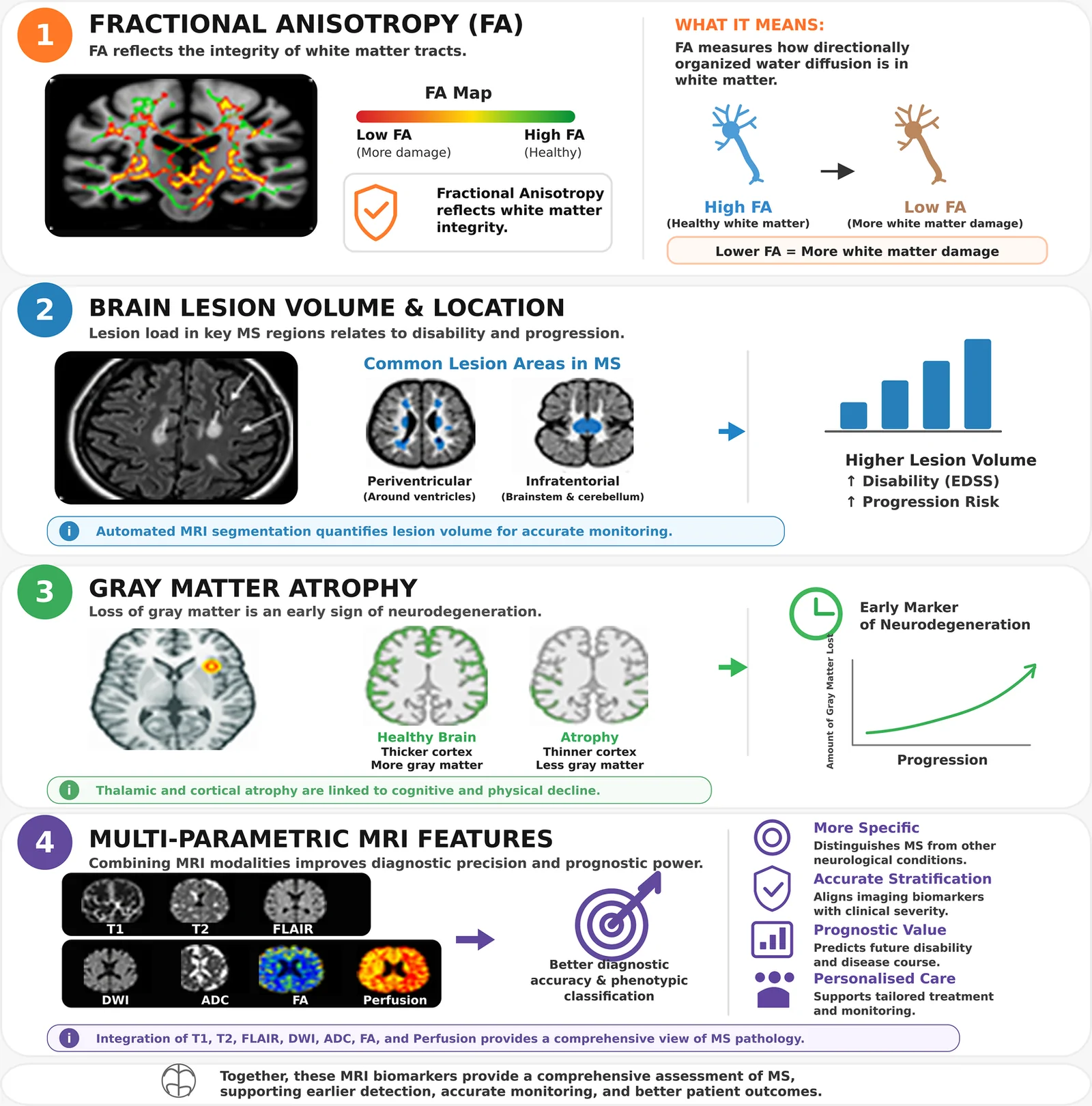
MRI-derived biomarkers and their relevance in MS diagnosis. Illustration of key MRI biomarkers: **(1)** FA representing white matter integrity; **(2)** lesion volume and location associated with disease burden and disability progression; **(3)** gray matter atrophy reflecting neurodegeneration; and **(4)** multi-parametric MRI features integrating T1-weighted, T2-weighted, fluid-attenuated inversion recovery (FLAIR), diffusion-weighted imaging (DWI), apparent diffusion coefficient (ADC), and perfusion imaging. Key observation: Integrating structural and microstructural MRI features improves diagnostic accuracy and disease characterization.

However, although these MRI metrics are informative, there are certain limitations to their use in diagnosing multiple sclerosis. Specifically, variability in MRI scanning protocols and post-processing procedures affects the consistency of findings, thereby limiting the applicability of MRI biomarkers. For instance, FA measures are highly dependent on diffusion parameters and may be misleading due to differences in MRI scanning protocols. Likewise, although the brain lesion volume correlates strongly with disability progression, it fails to reflect cortical lesions or subtle pathologies, limiting the sensitivity of MS identification. In addition, gray matter atrophy usually implies permanent tissue loss, making this metric less valuable for early interventions. Finally, while multi-parametric MRI is beneficial for diagnosing MS, it adds complexity to analysis and may introduce bias due to metric integration.

#### 3.2.2 Genomic data-driven biomarkers

Genomic biomarkers provide critical insights into the molecular mechanisms underlying MS, enabling improved diagnosis and patient stratification. Key genetic markers, such as HLA-DRB115:01* (risk) and HLA-A02:01* (protective), highlight the role of immune-related genetic susceptibility. Transcriptomic signatures, including lncRNAs and miRNAs, further capture dynamic disease processes such as inflammation and oxidative stress. Additionally, SNPs in immune-related genes and gene expression profiling support subtype classification and treatment response prediction. The integration of these multi-omics layers enhances the identification of biologically relevant patterns, enabling more precise and personalized MS diagnosis and prognosis as shown in Figure 5.

**Figure 5:**
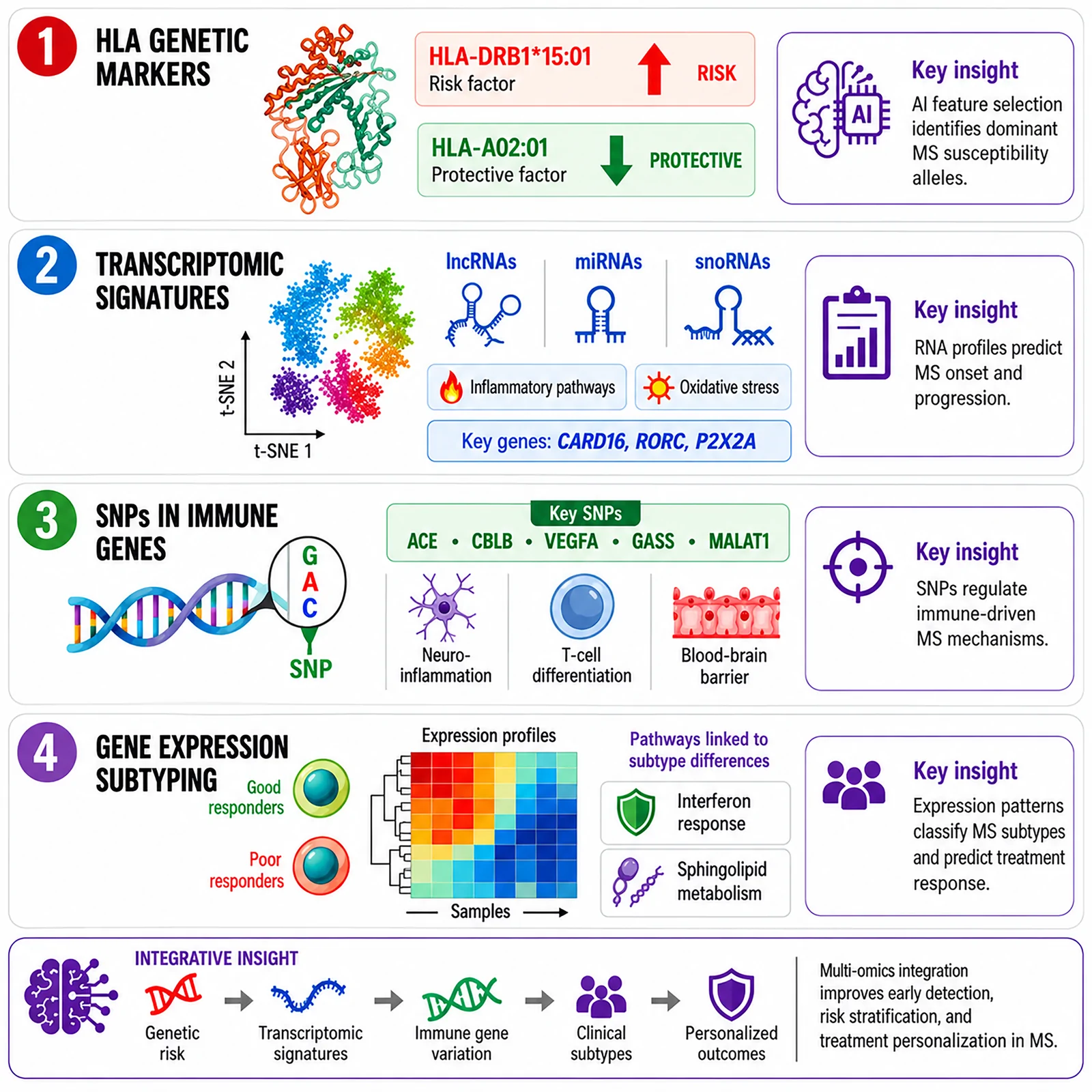
Genomic and transcriptomic biomarkers in MS. Overview of genomic biomarkers: **(1)** human leukocyte antigen (HLA) variants indicating genetic susceptibility; **(2)** transcriptomic signatures, including long non-coding RNA (lncRNA), microRNA (miRNA), and small nucleolar RNA (snoRNA), reflecting regulatory mechanisms; **(3)** single nucleotide polymorphisms (SNPs) capturing genetic variation; and **(4)** gene expression profiling for subtype classification and treatment response prediction. Key observation: Multi-omics integration improves risk stratification and supports personalized diagnosis.

While genomic biomarkers hold promise, they face certain challenges. High dimensionality and small sample sizes make overfitting likely and reduce reproducibility across populations. Population-specificity and external validation are common issues in SNP association studies and other discoveries, hindering the generalizability of results. Transcriptomic biomarkers also suffer from instability due to external factors and over time. In addition, although AI systems can identify statistically significant variables, separating causality from correlation can be difficult. Although multi-omics can increase accuracy, it also poses challenges, including integration bias.

#### 3.2.3 OCT biomarkers

As shown in Figure 6, OCT-derived retinal biomarkers are clinically feasible, non-invasive markers of neurodegenerative changes in multiple sclerosis. RNFL and GCIPL thickness measures represent axonal and neuronal damage, respectively, while inner nuclear layer changes and microcystic macular edema represent inflammation. Further information may be gained by assessing retinal vessel features. As the OCT collection process is relatively standardized, it enables reliable biomarker extraction, and using machine learning algorithms significantly increases their predictive power, enabling monitoring of disease progression. However, such biomarkers are fundamentally localized to the eye and the optic nerve and cannot capture extensive central nervous system pathology. In addition, sensitivity may be affected by prior optic neuritis. Furthermore, small, internally validated databases increase the risk of overfitting in many studies involving OCT imaging and AI models. Therefore, while OCT biomarkers are scalable and robust, their clinical usefulness is enhanced when used alongside additional imaging and genomic approaches.

**Figure 6:**
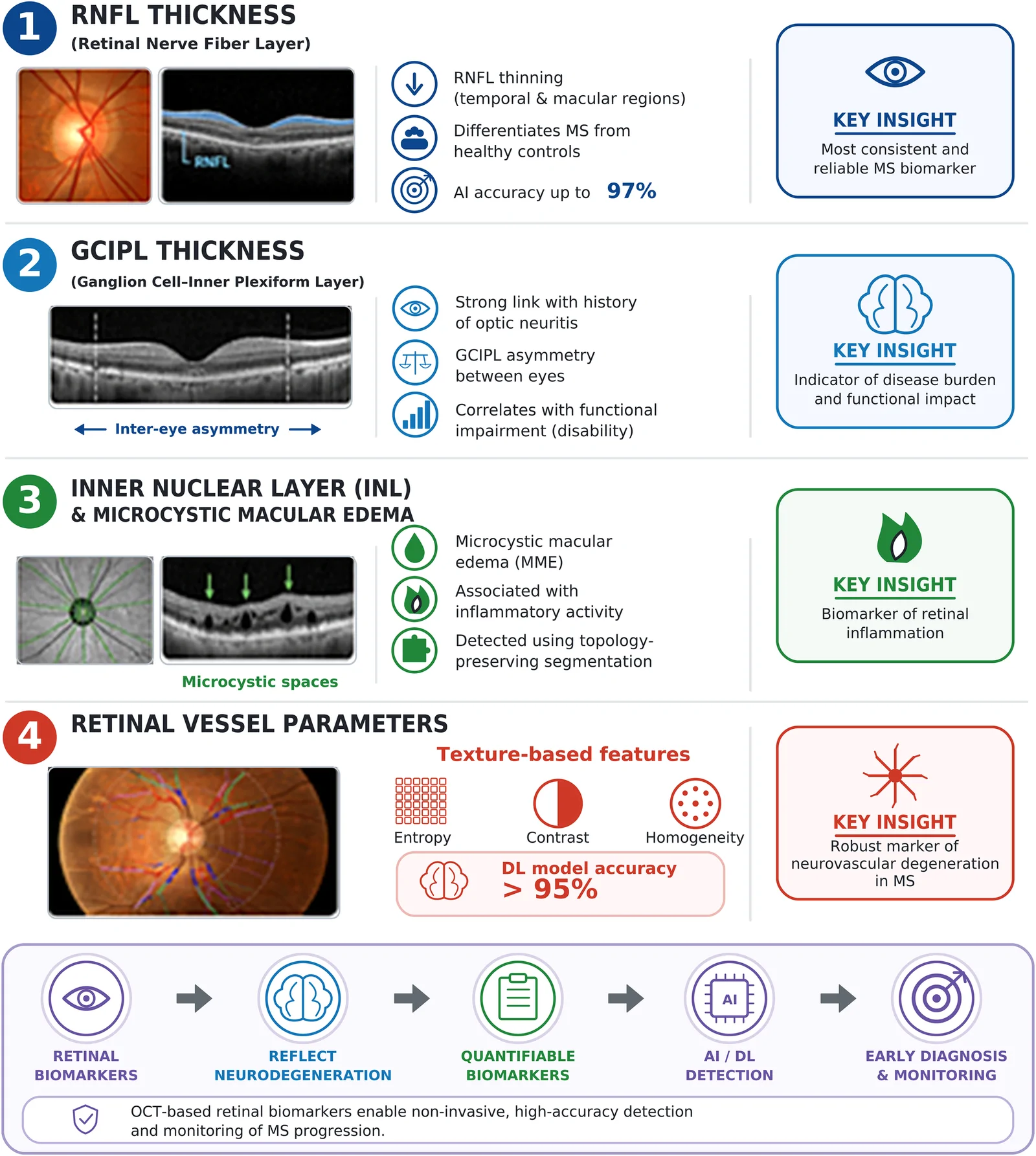
OCT-based retinal biomarkers for MS detection and monitoring. Illustration of retinal biomarkers: **(1)** retinal nerve fiber layer (RNFL) thickness indicating axonal loss; **(2)** ganglion cell–inner plexiform layer (GCIPL) thickness associated with neuronal damage; **(3)** inner nuclear layer (INL) changes and microcystic macular edema (MME) reflecting inflammatory processes; and **(4)** retinal vessel parameters capturing neurovascular alterations. Key observation: OCT provides non-invasive, high-resolution biomarkers for early detection and monitoring of MS progression.

#### 3.2.4 Other key biomarkers

Figure 7 highlights that combining clinical and functional biomarkers provides additional, necessary, and complementary information for diagnosing and prognosing MS patients compared with imaging and molecular biomarkers. The use of EDSS scales allows us to evaluate disease progression, while cytokines provide information about ongoing inflammation. On the other hand, functional tests reveal clinical significance by identifying impairments in the patient's vision and demonstrate excellent predictive performance. In addition, SNPs allow us to predict treatment efficacy. However, biomarkers are usually very variable and subjective. The use of EDSS scoring is prone to inter-rater bias, whereas cytokine levels depend on time- and environment-related factors. Even though functional tests may be clinically relevant, their specificity can be limited. Therefore, incorporating such biomarkers into multimodal artificial intelligence models is crucial.

**Figure 7:**
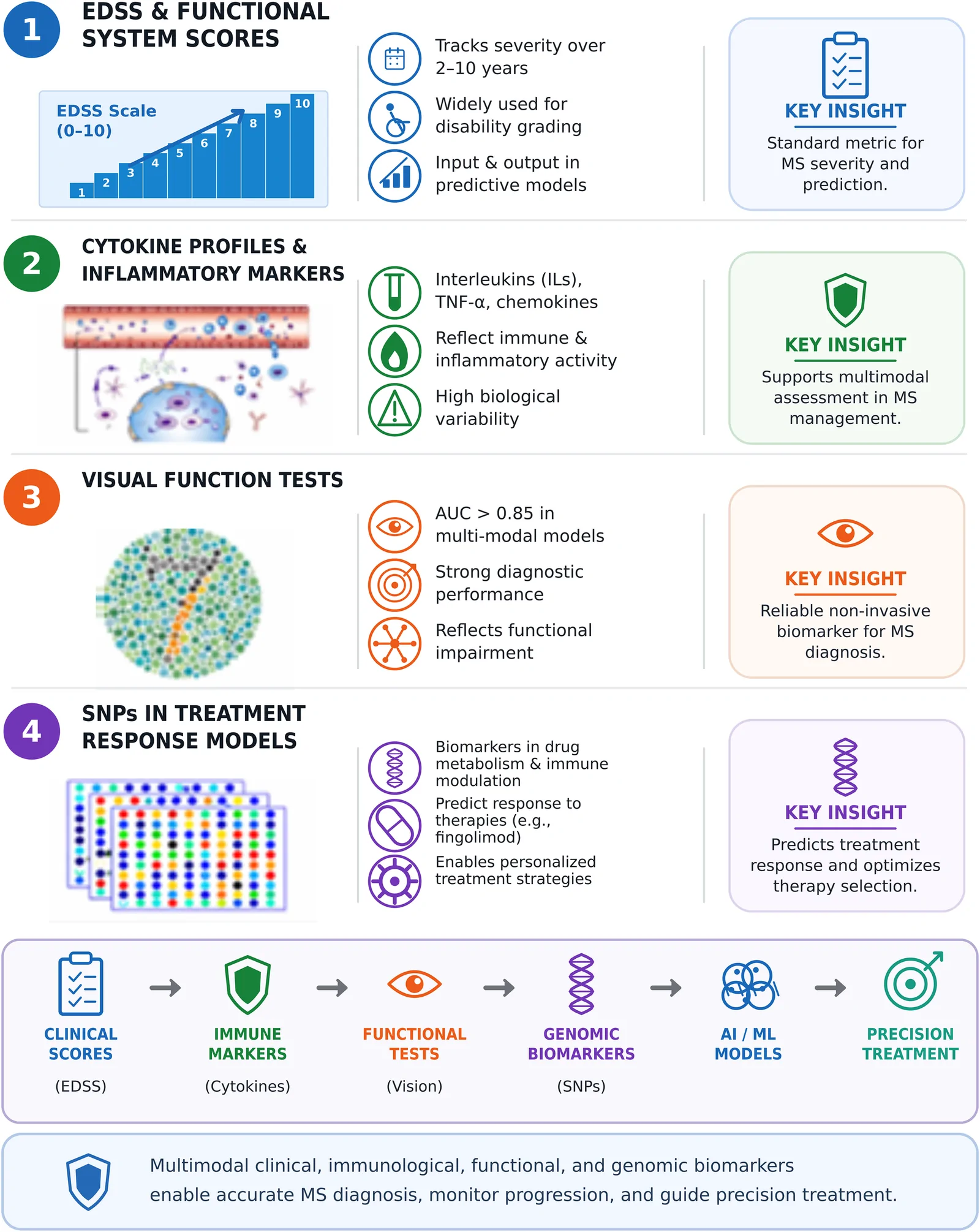
Complementary clinical, functional, and immunological biomarkers in MS. Depiction of non-imaging biomarkers: **(1)** Expanded Disability Status Scale (EDSS) quantifying disease severity; **(2)** cytokine profiles [e.g., interleukins, tumor necrosis factor-α (TNF-α)] reflecting immune activity; **(3)** visual function tests indicating functional impairment; and **(4)** SNP-based biomarkers predicting treatment response. Key observation: Integration of clinical and biological markers enhances disease monitoring and personalized treatment strategies.

#### 3.2.5 Comparative analysis of AI models across modalities in MS diagnosis

The cross-modal comparison of AI models in Table 6 shows that an AI model's quality depends not only on its choice but also on the input data and methodology. In MRI-based studies, CNNs tend to perform better because of their capability to learn more complex spatial patterns associated with lesion localization and brain atrophy. However, these models’ performance is often adversely affected by methodological issues, including small sample sizes (fewer than 200 participants), insufficient validation, and possible data leakage during pre-processing. While genomic studies rely on RF models and ensembles to process high-dimensional data and identify relevant gene signatures, the mismatch between data dimensionality and sample size makes them susceptible to overfitting and population-related bias. Likewise, OCT-based models, particularly SVMs and RFs, perform better due to standardized imaging and well-defined features, including RNFL thickness. However, these models do not fully capture disease complexity.

**Table 6:** Model–modality comparative matrix for AI-based MS diagnosis.

Modality	Dominant models	Why they perform well	Key limitations/Failure modes
MRI	CNNs, U-Net, RF	CNNs capture spatial lesion patterns and brain structure; RF is effective with radiomics.	Small datasets (*n* = 100–180), no external validation, data leakage risk in preprocessing, variability in MRI protocols
Genomics	RF, Ensemble ML, ANN	Handles high-dimensional molecular data; identifies disease-related gene signatures	Overfitting due to high dimensionality, population bias, lack of biological validation, and weak generalizability
OCT	RF, SVM, ML classifiers	Standardized acquisition: stable features; strong RNFL biomarkers	Limited to retinal pathology, weak system-level insight, mostly internal validation, and limited interpretability
Multimodal	RF, Ensemble, Hybrid models	Integrates complementary imaging + molecular + clinical data: better generalization	Data harmonization issues, missing modalities, increased complexity, reduced interpretability, and small datasets

This table compares dominant AI models across modalities, including MRI, genomics, OCT, and multimodal approaches. It summarizes model types (e.g., CNNs, Random Forest, SVM), their performance advantages, and key limitations such as overfitting, data heterogeneity, and limited interpretability. Key observation: Model effectiveness depends on modality-specific characteristics, with trade-offs between accuracy, generalizability, and interpretability influencing clinical applicability.

Despite these limitations, multimodal methods have shown promising results by combining several data types into a single model, yielding performance gains and greater consistency. At the same time, their drawbacks include higher complexity, difficult data harmonization, and reduced explainability. In addition, multimodal models suffer from missing data across diverse datasets and from small dataset sizes.

The reported performance values should not be interpreted as direct indicators of superiority between modalities or algorithms. Substantial heterogeneity exists across the included studies in cohort size and composition, disease phenotype, control-group definition, data acquisition and preprocessing, validation strategy, and diagnostic or prognostic endpoint. Some studies rely primarily on internal cross-validation, whereas others use multicenter cohorts or other forms of independent validation. The reported metrics also represent different tasks, including disease classification, lesion or tissue reconstruction, disability prediction, prognosis, and treatment-response estimation. Consequently, differences in accuracy, sensitivity, specificity, or related measures may reflect methodological and endpoint variability as much as intrinsic model performance. Cross-study comparisons in this review should therefore be interpreted as descriptive rather than as direct head-to-head evidence of diagnostic superiority.

#### 3.2.6 Interpretability, reliability, and clinical utility

Interpretability in the context of AI-based MS diagnosis is an important yet understudied factor in these studies. Even though some studies implement explainable AI (XAI) techniques, such as LIME for *post-hoc* feature attributions, none assess the stability or clinical relevance of these explanations. For example, Ghafouri-Fard et al. (56) apply LIME to SNP-based MS classification. However, the method is sensitive to the choice of neighborhood, sampling strategy, and other factors, raising concerns about its reproducibility. In studies that rank biomarkers according to importance in RF, such as those by Acquaviva et al. (53) and Omrani et al. (54), only global interpretations are provided. However, even though these models have higher explanatory power, they do not necessarily imply causal relations or clinical significance, especially when there are many input features, where correlations can affect importance estimates.

In imaging studies, particularly OCT-based ones, researchers often assume that using relevant features, such as RNFL thickness or lesion location, implies interpretability because of their anatomical relevance, but this is not enough to uncover the deep-learning model's reasoning process. At the same time, methods such as visualizing activations with saliency maps or applying an attention mechanism are less frequently reported, not standardized, and not validated for consistent results.

There is a trade-off in deep learning between interpretability and performance as better performance is achieved at the expense of interpretability in terms of stability and reproducibility. Simple models are more interpretable since they produce fewer complex interactions and are less likely to fit disease heterogeneity. However, the problem with current approaches to interpretability is that none of the studies evaluate the effect of interpretations on physician confidence and decision-making.

One of the main challenges in developing deep learning interpretation tools is the trade-off between model complexity and interpretability. Since such models achieve higher accuracy by incorporating more complex representations of the input data, their explanations tend to be less stable and difficult to interpret. On the other hand, a simpler architecture might offer greater clarity but may not capture the complexity of diseases. However, no studies have evaluated how explanations affect clinicians' trust and decision-making.

### 3.3 RQ3: emerging trends and multimodal integration

Future research should focus on real-time clinical trials that integrate AI models into the operations of healthcare facilities and diagnostic centers. XAI frameworks should be the primary focus of future research (75–77). These frameworks should provide clinically interpretable decision-support tools and emphasize the rationale behind specific predictions (78). To bridge the gap between AI model predictions and human expertise, techniques such as attention mechanisms, saliency maps, and feature significance analysis could be used. Regulatory approval procedures are necessary for broad implementation of AI models in clinical practice. Future research should focus on frameworks for artificial intelligence ethics, data governance, and algorithmic fairness.

To facilitate real-time clinical decision-making, research should investigate approaches for smoothly incorporating AI technologies into EHRs. Unlike monitoring disease progression over time, most AI models rely on static, baseline data. To enable dynamic, real-time MS diagnosis, future research should focus on developing AI models using longitudinal data. AI-driven techniques may use polygenic risk scores to categorize patients by genetic vulnerability. This strategy can initiate early intervention for those at high risk. The emergence of novel AI methods has significantly impacted the process of biomedical data analysis, particularly within fields that involve high-dimensional and multimodal data analysis like MS. While standard AI approaches have been successful in classification problems related to imaging and genomics, newer approaches like foundational models, multimodal representation learning, and causal AI have expanded the possibilities of AI-based diagnostics.

#### 3.3.1 Federated learning approaches

Using multi-parametric MRI and AI-based FA reconstruction, some studies have demonstrated the ability to differentiate between MS-related pathologies. These findings largely relied on institutional data, limiting their applicability. They have shown promise in predicting MS onset, phenotype, and disease progression using transcriptome and SNP data. Privacy laws, such as the General Data Protection Regulation (GDPR) and the Health Insurance Portability and Accountability Act (HIPAA), often restrict sharing genetic data across organizations (79–83). To develop AI models applicable to a wide range of individuals, massive, standardized, multi-center datasets encompassing medical, imaging, and genetic information are needed (84–86). To address RQ3, federated learning enables multiple healthcare centers to train a model without sharing an individual's sensitive data. For instance, domain-specific data normalization and augmentation may be used to train federated CNNs architectures across institutions (87–89). This improves cross-center applicability without compromising patient privacy, enabling consistent lesion segmentation, brain volume measurement, and MS subtype categorization. Federated learning enables models to be trained across clinics using various OCT systems (Spectralis, Cirrus, Topcon) and diverse patient populations at different disease stages. Personalized federated learning algorithms and cross-device harmonization layers enable the adaptation of model weights to local device features while sharing global patterns. Based on retinal imaging, this strategy will provide a scalable, privacy-preserving, and clinically interpretable AI-based MS diagnosis.

#### 3.3.2 Foundation models and transfer learning in biomedical AI

The term foundation models (FMs) denotes a paradigm shift from specialized AI to generalist models in biomedical applications, with the capacity to learn a wide range of downstream tasks under minimal supervision from large pre-trained systems. In the domain of medical imaging and genomics, these FMs are now advancing precision medicine by using extensive unlabeled data from imaging or omics sources through self-supervised learning (87, 88). In line with recent research directions (90, 91), imaging FMs use multimodal architectures that incorporate modalities such as CT, MRI, ultrasound, histopathology, and three-dimensional imaging within a single framework.

Contemporary progress in these models involves attention-based fusion approaches and graph-based integration to account for complex connectivity patterns that prove especially useful in conditions like multiple sclerosis (92). The trend toward zero-shot and few-shot learning reduces reliance on large, annotated training sets, while initiatives to create fair and equitable FMs aim to minimize demographic and institutional biases (93, 94). From a future developments standpoint, several critical trends include federated foundation models that enable privacy-preserving collaborations between institutions, the use of causal inference models, and the design of reliable, transparent FM architectures appropriate for clinical settings (94). Moreover, FMs are expected to integrate patients' lifestyles, environments, and other multimodal data to enable hyper-personalized medicine.

Despite these advantages, several challenges remain, including computational demands, interoperability, data heterogeneity, interpretability, and ethical considerations in using FMs. Interdisciplinary cooperation and innovations in these areas are crucial for advancing the clinical applications of FMs to a new level. Overall, the concept of FMs has the potential to transform biomedical imaging and genomics.

#### 3.3.3 Multimodal representation learning

Multimodal representation learning has become the principal paradigm for fusing heterogeneous biomedical data, including MRI, OCT, genomic data, and clinical features. Unlike unimodal learning, multimodal models learn a shared latent representation that leverages different information across inputs (95–97). The field of multimodal representation learning remains dynamic, with ongoing advances driven by increasingly advanced fusion methods that combine different modalities without sacrificing temporal dynamics or cross-modal correlations. Recent studies reveal three novel paradigms that address fundamental drawbacks of previous methodologies.

One recent innovation has been the dual fusion attention mechanism (97). The dual-component model uses a multimodal fusion attention module to generate enhanced representations from diverse sources, including dermoscopic images, Pap smears, magnetic resonance imaging, and computed tomography. It then uses a multimodal information fusion attention component to derive representations that capture common features of interest. When embedded in a deep learning network, it achieves impressive results on medical imaging benchmark tests and employs Monte Carlo Dropout ensembles for uncertainty quantification (97).

Benchmarking methods have become another essential tool for evaluating multimodal algorithms. MultiBench offers a comprehensive, large-scale benchmarking platform covering 15 datasets, 10 modalities, 20 prediction tasks, and 6 research areas (including health care). It evaluates algorithm performance on generalization, computational efficiency, and sensitivity to noisy or missing modalities. It also promotes reproducibility and accelerates development towards deployment in practical applications (98).

Temporal data can be processed through the Correlational Recurrent Neural Network (CorrRNN). The CorrRNN jointly learns representations and temporal structures. It includes several loss terms, such as maximizing correlation, and an adaptive attention mechanism that assigns different weights to modalities at each step of the sequence. Applications include video, audio, and sensory signals, yielding state-of-the-art accuracy in activity recognition and audio-visual speech synthesis tasks (97, 98). Collectively, these techniques—dual attention fusion, standardized multiscale benchmarking, and correlation-aware temporal modeling—represent the frontier of multimodal representation learning. They pave the way for more robust, interpretable, and clinically translatable systems, particularly in precision medicine.

#### 3.3.4 Explainable and causal AI for biomarker discovery

Explainability and causal inference have emerged as crucial aspects of modern biomedical artificial intelligence for biomarker discovery in fields such as oncology, neurology, and precision medicine (99). ML models are often described as black boxes that can make accurate predictions but lack transparency in their decision-making processes. This issue limits clinicians' understanding, poses regulatory approval challenges, and impedes the use of the resulting data for therapy development. To address these shortcomings, several explainability approaches, such as SHapley Additive exPlanations (SHAP), Local Interpretable Model-Agnostic Explanations (LIME), and attention mechanisms, have been proposed to enable interpretable outputs that allow clinicians to estimate biomarker significance and identify potential biases (100).

In addition to interpretability, causal inference allows for distinguishing between causation and correlation, which is especially important given the high dimensionality of biomedical datasets (101–103). High dimensionality makes omics data vulnerable to confounders and selection bias, leading to unreliable biomarker discovery when predictive modeling is used alone (104). However, techniques such as causal graphs, propensity score matching, and counterfactual analysis enable discovery of biologically relevant mechanisms driving disease or treatment response. Several studies show that integrating causal inference into machine learning improves the stability and reproducibility of biomarker identification. The combination of both explainability and causal inference in the form of causal explainable artificial intelligence (Causal XAI) could contribute to the reliability of biomarker discovery since these two techniques complement each other by bringing the advantages inherent to the field of causality as well as interpretability to the field of prediction (101, 103, 105–108). Finally, applications across various fields of biomedicine show that these hybrid methodologies can uncover relationships not possible with correlation-based models while enabling model validation and regulatory compliance.

#### 3.3.5 Information-theoretic feature selection and high-dimensional learning

The information-theoretic feature selection (ITFS) method is a mathematically rigorous and computationally efficient filter-based procedure that uses fundamental information-theoretic concepts, such as entropy, mutual information, conditional entropy, and interaction terms, to select relevant yet redundant features in ultrahigh-dimensional datasets. Unlike the wrapper and embedded approaches, which require a particular classifier, ITFS is not tied to any model and can therefore be applied to datasets that would otherwise render these techniques computationally intractable (e.g., RNA-seq, metagenomics, and genetic risk profiling).

The key idea behind ITFS is to maximize mutual information between each feature and the target variable (relevance) while minimizing mutual information between selected features (redundancy). While classical criteria, like minimum redundancy–maximum relevance (mRMR), remain useful, there have been modern updates to the concept: for example, copula entropy can be used to consider linear and non-linear interactions in high-dimensional genetic data sets regardless of any Gaussian distribution, and therefore enables interactive feature selection (106). Such modern methods perform exceptionally well in high-dimensional learning, where the number of predictors (*p*) exceeds the number of observations (*n*), leading to the “curse of dimensionality,” overfitting, and spurious correlations.

High-dimensional learning refers to the difficulties encountered by classical statistical models in situations where *p* ≫ *n*, leading to a variety of problems, including the breakdown of distance measures and increased likelihood of capturing random correlations. The strength of ITFS lies in its ability to serve as an intermediate step in feature selection, reducing data dimensionality while preserving biologically meaningful signals and removing noise. In practice, it will improve model generalizability, reduce computational costs for subsequent modeling, and improve explainability (109–111). This technique has demonstrated numerous advantages in recent literature. For example, hybrid causal feature selection integrates information-theoretic criteria with causal graph algorithms to identify relevant biomarkers in RNA-seq studies (106).

Copula-entropy-based feature selection outperforms traditional MI methods for high-dimensional genetic data (112). Copula-entropy-based methods outperform traditional MI approaches in high-dimensional genetic data by modeling complex dependencies (112). Information-theoretic pre-filtering combined with machine learning yields more reliable genetic risk prediction models (113), and strategic feature selection pipelines enable effective deep-learning classification even in ultra-high-dimensional genomic datasets (114). Overall, ITFS provides a mathematically grounded, scalable solution that bridges high-dimensional learning challenges with clinically actionable biomarker discovery.

### 3.4 Unified multimodal AI framework for MS diagnosis and prognosis

Beyond technical optimization, developing an MS diagnosis framework must also consider clinical perspectives to ensure data and models are clinically relevant and socially useful. Domain knowledge is essential for selecting suitable biomarkers, defining meaningful endpoints (e.g., disease progression or scores), and ensuring that model outputs are usable and reflect real-life decision-making processes. Public health perspectives play a significant role in recognizing variability across populations, including disease prevalence, environmental risks, and healthcare availability. Insights from the social sciences help in understanding how patient behavior, health literacy, and trust can affect AI adoption. These perspectives directly influence data selection, model development, and evaluation, emphasizing the need for representative datasets, clinically relevant features, and evaluation metrics that extend beyond accuracy to include fairness and utility. Integrating these dimensions supports the development of AI systems that are not only technically robust but also clinically meaningful and socially responsive.

Methodologically, using diverse, representative datasets reduces the risk of algorithmic bias, while domain-expert-informed feature selection improves model interpretability. Evaluation criteria should include fairness and robustness metrics in addition to traditional accuracy measures. Combining such aspects ensures that the resulting models are technically and ethically sound for implementation in clinical settings. Therefore, including the mentioned dimensions will enable the development of AI systems that combine high technical performance with clinical validity and ethical compliance. Consequently, a framework for reliable MS diagnosis is recommended, including layers dedicated to interpretability, validation, and bias prevention.

The unified framework (Figure 8) compiles major methodological trends identified in the included studies, providing an organized pipeline for integrating heterogeneous data sources in MS diagnosis and prognosis. It starts with multimodal data acquisition, including MRI, OCT, genomic, clinical, and EHR data, to capture complementary aspects of disease pathology. These inputs are preprocessed and feature-extracted using modality-specific methods to identify significant biomarkers, including lesion features, retinal layer measurements, and gene expression. These features are then integrated into a single representation using multimodal methods, enabling the modeling of more complex cross-modal relationships. Machine learning, deep learning, and ensemble models are then used for classification and prediction. In line with the reported study limitations, a validation and bias-control layer improves robustness to external validation and bias. Lastly, interpretability approaches enable clinically actionable results in diagnosis, progression forecasting, and treatment stratification.

**Figure 8:**
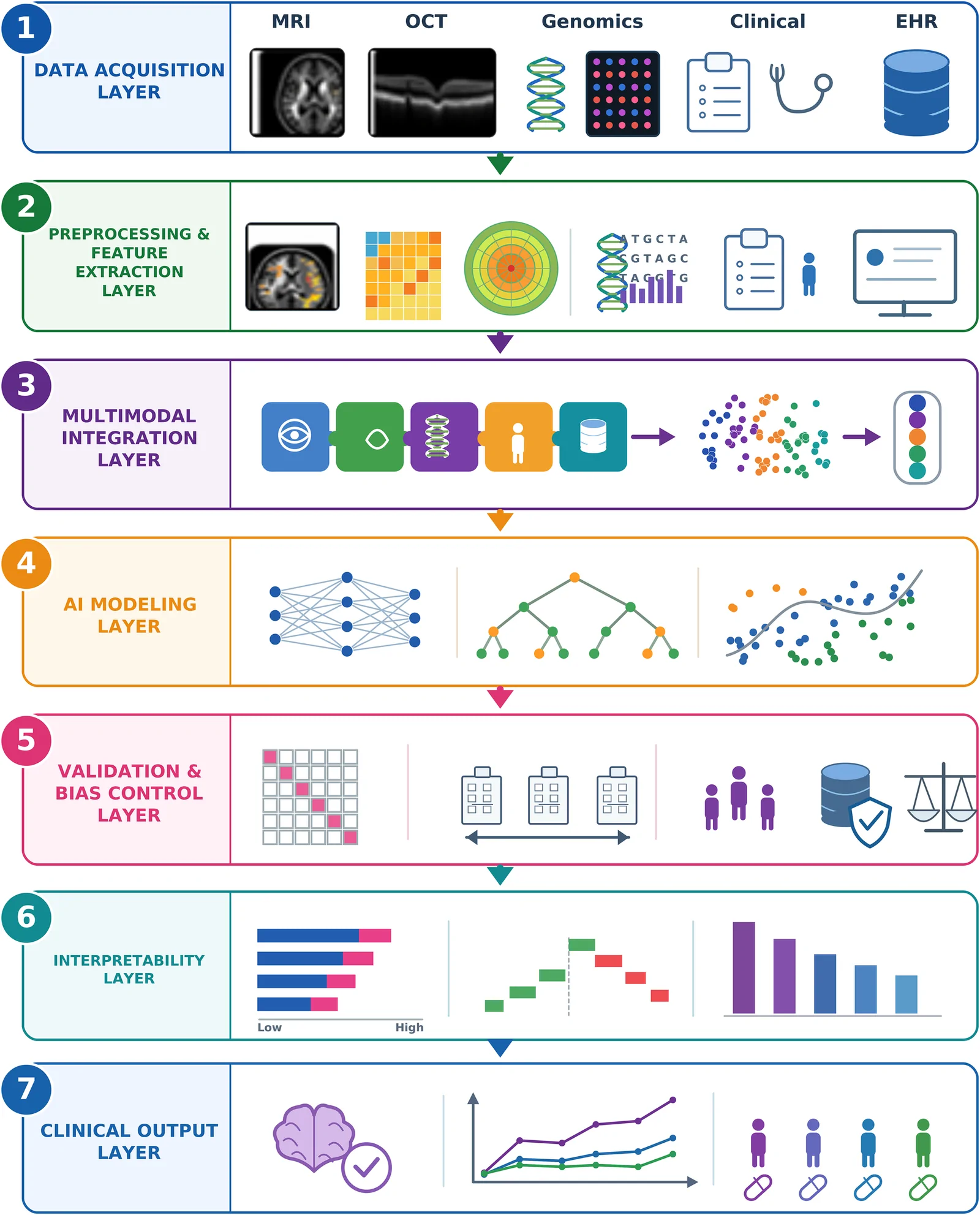
Generalized multimodal AI framework for MS diagnosis. Schematic representation of the AI pipeline: **(1)** data acquisition from MRI, OCT, genomics, clinical data, and EHR; **(2)** preprocessing and feature extraction; **(3)** multimodal integration via feature fusion; **(4)** AI modeling using ML and deep learning; **(5)** validation and bias control, including cross-validation and cohort-level assessment; **(6)** interpretability using explainable AI methods such as SHAP and LIME; and **(7)** clinical outputs, including diagnosis, progression prediction, and treatment stratification. Key observation: Robust validation and interpretability are critical for clinical translation.

## 4 Discussions

This review primarily contributes by providing a thorough analysis of AI-based research that uses medical imaging, genomic data, or multi-source clinical data to diagnose multiple sclerosis. According to the study findings, AI techniques, including ML and DL approaches, extract hidden patterns and key biomarkers from complex datasets. Compared to traditional diagnostic methods, AI-driven approaches provide superior predictive capabilities. This review provides a valuable foundation for further investigation and the development of AI-powered MS diagnostic tools. This synthesis highlights promising indicators, including RNFL/GCIPL thinning, lesion volume, transcriptomic markers, and SNPs. Researchers can prioritize clinically essential characteristics to develop effective models. This study summarizes ML and DL methodologies, evaluation criteria, and limitations. To improve diagnostic accuracy and personalization, it emphasizes the benefits of integrating multiple data sources, including imaging, clinical, and genetic information. Additionally, it highlights federated learning as a potential avenue for future study to address privacy concerns and improve the model's generalizability across diverse clinical settings. Based on these findings, future AI models will be more likely to prioritize ethics, privacy, and collaboration. To enhance the reproducibility, comparability, and impact of future research, the review findings promote the development of standardized evaluation criteria, validation processes, and data-sharing frameworks.

While many studies report high diagnostic performance, often exceeding 90%, focusing solely on accuracy can overlook important ethical considerations in AI-based MS diagnosis. As emphasized in the study (107), model evaluation should shift from a single-objective focus to a multi-objective approach that balances accuracy, fairness, privacy, and trust. For instance, high model accuracy is often achieved with homogeneous training sets; however, the same model can produce biased or unfair results when generalizing to different patient groups due to insufficient variation in demographic or clinical features. Despite frequent reports of high accuracy, these findings should be interpreted with caution. Many studies rely on relatively small sample sizes (often <200 subjects), increasing the risk of overfitting and limiting statistical robustness. Additionally, most models are validated using internal strategies, such as cross-validation, with limited use of independent external cohorts, thereby limiting generalizability to real-world clinical settings. Dataset homogeneity, particularly the predominance of European populations, further constrains applicability across diverse patient groups. Therefore, the reported performance reflects research-level optimization rather than clinically deployable systems, underscoring the need for large-scale, diverse, and externally validated studies.

Interpretability builds clinicians' trust only when the interpretation is actionable along clinical pathways, not only an algorithm's output. With current developments in MS research, explanation techniques have been rarely adopted in clinical practice for radiological reporting, treatment selection, or long-term patient monitoring. To be effective and interpretable, outputs must refer to tangible objects recognized in clinical contexts, such as lesion location, lesion progression patterns, and reliable biomarkers, and be presented in a form consistent with the interface used for disease diagnosis. Moreover, building trust requires more than a clear rationale; it requires consistency and accountability, ensuring that similar patient profiles receive the same interpretation at any time. Clinical explanations should also be offered not to justify decisions already made but to support decision-making when no obvious course of action exists.

In current assessments of AI models used for MS diagnosis, the emphasis remains heavily skewed toward predictive performance and accuracy. Other ethical considerations, such as fairness, robustness, and patient data privacy, are often overlooked. While AI models often achieve high predictive accuracy, this does not ensure consistent performance across populations or guarantee the absence of bias. Highly optimized models can limit their applicability when deployed in new environments, perpetuating existing health inequities and discrimination. Thus, re-evaluating current AI assessment criteria is necessary to introduce multi-objective criteria and balance predictive accuracy with other considerations.

Moreover, using high-dimensional imaging and genomic data raises serious privacy and confidentiality concerns that must be addressed. In addition, multimodality increases these risks since the information used is sensitive and includes a variety of health data. Over-optimizing algorithms for specific patient types also becomes problematic, potentially undermining a machine learning algorithm's ability to generalize and reducing disease variance. Thus, future research should consider ethical implications when assessing model performance.

Current AI systems used to predict MS presence primarily focus on biological indicators, such as genomic data and neuroimaging scans, while largely ignoring other factors that significantly impact patient health and disease development. Lifestyle-related variables, such as smoking habits or vitamin D exposure; environmental factors, including location and sunlight exposure; and socio-economic status are all significant factors affecting disease development (113). However, their importance is not sufficiently considered by AI algorithms. Ignoring these factors may lead to biased, imprecise models with poor generalization across populations. Integrating such factors into multimodal AI models yields a more comprehensive representation of patients, thereby improving prediction quality and enabling personalized approaches (111–113). Moreover, considering these factors helps identify vulnerable groups and supports an inclusive approach to medical care.

AI-powered MS diagnosis often relies heavily on simple demographic variables, such as age or sex, without taking into account intersectionality—a combination of different socio-biological factors affecting the health condition simultaneously (114). The reductionist approach ignores the reality that ethnicity, socio-economic status, geography, and health care access collectively affect disease outcomes and the effectiveness of the diagnostic model being utilized (115). Models built on homogeneous datasets or a narrow selection of subgroups may introduce biases that can lead to underdiagnosis or misdiagnosis in less-represented groups. Intersectional fairness in AI aims to solve this problem through subgroup evaluation and fairness-aware learning algorithms.

A key limitation is the reliance on coarse demographic categorizations, which fail to capture nuanced population heterogeneity and may obscure subgroup-specific risks (116). Emerging approaches in intersectional fairness aim to address these gaps by evaluating model performance across intersecting subgroups, incorporating fairness-aware learning objectives, and leveraging stratified or reweighted training strategies. Integrating these methods is essential for developing equitable, generalizable, and clinically reliable AI systems in MS diagnosis. Existing evaluations of ML algorithms for diagnosing MS have primarily focused on accuracy, neglecting important ethical implications related to fairness, privacy, and equity (116). Although higher accuracy does not necessarily mean more balanced performance, training AI on imbalanced data can lead to biased predictions and unequal patient outcomes, especially if the dataset lacks diversity in demographics and socioeconomic factors. Another key limitation is over-optimizing models for narrowly defined sub-populations, which may inflate performance metrics while reducing generalizability across diverse clinical settings (117). A balanced ethical perspective requires incorporating fairness-aware evaluation, bias mitigation strategies, and privacy-preserving techniques to ensure that AI systems are not only effective but also equitable and trustworthy in real-world clinical practice.

Although the review employs a comprehensive approach to identifying studies on AI-based MS diagnosis across the genome, medical imaging, and other modalities, it has certain limitations. The methods, imaging procedures, and genetic analysis techniques used in the evaluated studies differ, making direct comparison of the findings difficult. MRI data acquisition and AI model training and evaluation are not standardized. This inconsistency leads to findings that are not repeatable across medical settings, limiting their generalizability. The authors identify relevant research systematically. Although the study offers a comprehensive overview of AI-powered MS diagnosis, certain limitations may influence its outcomes. The dependency on peer-reviewed research articles may have overlooked potential insights from preprints, conference proceedings, and institutional reports. Restricting the review to English may have introduced linguistic bias. Consequently, the review may have excluded valuable research from non-English-speaking countries.

Additionally, selection bias may have occurred despite the review's use of several significant databases. In particular, these databases may have missed AI-driven research published in multidisciplinary or domain-specific venues, with significant consequences for MS diagnoses, that are not indexed in reputable databases. The heterogeneity among the included studies is another limitation. The diversity of assessment measures made direct comparisons and quantitative synthesis via meta-analysis infeasible. This heterogeneity limits the interpretation of performance benchmarks and the generalizability of findings. Finally, most included studies prioritized point-in-time diagnosis over long-term illness monitoring. This evaluation cannot conclude the use of AI for disease trajectory or treatment response prediction in multiple sclerosis because no longitudinal or prognostic models are available. To support the clinical use of artificial intelligence in comprehensive MS treatment, future studies must address these shortcomings.

## 5 Conclusions

In advancing MS diagnosis, this systematic review highlights AI's multifaceted contributions through the integration of medical imaging, genetic, and diagnostic data. The reviewed studies indicate that AI-powered models have significant potential to improve diagnostic accuracy, enable early disease detection, and support personalized risk stratification. Artificial intelligence models have effectively extracted key structural biomarkers from medical imaging, including MRI and OCT. These biomarkers offer a non-invasive, objective method for detecting neurodegenerative changes, particularly in the early or subclinical stages of MS. By integrating transcriptomic signatures, genetic studies have enriched the diagnostic landscape. AI models showed stronger potential to personalize diagnosis and care when combined with clinical factors such as EDSS scores, recurrence rates, and treatment responses. To a significant extent, the study emphasizes the importance of integrating multiple modalities to improve MS diagnostic performance. However, the study highlights ongoing challenges, including the model's lack of transparency, limited external validation, and data heterogeneity. Developing reliable, scalable MS diagnostic tools requires a strong focus on explainable AI, multimodal integration, and federated learning. To guarantee accurate, individualized, and equitable treatment, these guidelines are crucial for transitioning AI-based MS diagnoses from research to real-world clinical practice. Although this review provides valuable insights into AI applications for diagnosing MS, it has certain limitations. In addition to language and publication bias, excluding non-English publications and non-peer-reviewed sources may have omitted emerging research. Future evaluations should include a wider range of data sources, such as preprints and literature in languages other than English, and adopt standardized techniques to improve comparability.
