# Supplementary material for: Artificial intelligence-driven multiple sclerosis diagnosis using genomic and medical imaging data: a review
Source: Front Med (Lausanne). 2026 Oct 29;13:1812134. doi: 10.3389/fmed.2026.1812134 (PMC13757027; doi:10.3389/fmed.2026.1812134)
Supplement: Supplementary file 1 [file Table1.docx]

**Supplementary Table S1: Risk of Bias Assessment (QUADAS-2)**

| **Study** | **Patient Selection** | **Index Test** | **Reference Standard** | **Flow & Timing** | **Overall Risk** |
| --- | --- | --- | --- | --- | --- |
| Huang et al. (2021) | High | Low | Low | Unclear | Medium |
| Taloni et al. (2022) | High | Low | Low | Low | Medium |
| Gaviraghi et al. (2022) | Medium | Low | Low | Unclear | Medium |
| Acquaviva et al. (2020) | Medium | Low | Low | Low | Low |
| Ghafouri-Fard et al. (2020) | Medium | Medium | Low | Low | Medium |
| Fuh-Ngwa et al. (2022) | Low | Low | Low | Low | Low |
| Jeyananthan (2024) | High | Medium | Low | Unclear | Medium |
| Omrani et al. (2024) | Medium | Low | Low | Low | Low |
| Arnal Segura et al. (2025) | Medium | Medium | Low | Unclear | Medium |
| Montolío et al. (2021) | Medium | Low | Low | Low | Medium |
| Pérez del Palomar et al. (2019) | Medium | Low | Low | Low | Medium |
| He et al. (2019) | High | Low | Low | Unclear | Medium |
| Khodabandeh et al. (2024) | Medium | Low | Low | Low | Low |
| Ciftci Kavaklioglu et al. (2022) | Medium | Medium | Low | Low | Medium |
| Ortiz et al. (2023) | Medium | Low | Low | Low | Medium |
| Arian et al. (2023) | Medium | Medium | Low | Unclear | Medium |
| Aghababaei et al. (2024) | Medium | Low | Low | Low | Medium |
| Montolío et al. (2024) | Medium | Low | Low | Low | Medium |
| Dongil-Moreno et al. (2024) | Medium | Low | Low | Low | Medium |
| Kenney et al. (2022) | Low | Low | Low | Low | Low |
| Cavaliere et al. (2019) | Medium | Low | Low | Low | Medium |
| Yaghoubi et al. (2023) | Medium | Medium | Low | Unclear | Medium |
| Andorra et al. (2024) | Low | Low | Low | Low | Low |
| Pinto et al. (2020) | Medium | Medium | Low | Low | Medium |
| Pellegrini et al. (2020) | Medium | Medium | Low | Unclear | Medium |
| Ferrè et al. (2023) | Medium | Medium | Low | Low | Medium |
