# Supplementary material for: Artificial intelligence-driven multiple sclerosis diagnosis using genomic and medical imaging data: a review
Source: Front Med (Lausanne). 2026 Oct 29;13:1812134. doi: 10.3389/fmed.2026.1812134 (PMC13757027; doi:10.3389/fmed.2026.1812134)
Supplement: Supplementary file 2 [file Table2.docx]

**Supplementary Table S2: Comparative Methodological Assessment and Scoring**

| **Study** | **Modality** | **Dataset Size** | **Validation** | **External Validation** | **Interpretability** | **Score** |
| --- | --- | --- | --- | --- | --- | --- |
| Huang et al. (2021) | MRI | 116 | CV | No | No | 2 |
| Taloni et al. (2022) | MRI | 181 | CV | No | No | 2 |
| Gaviraghi et al. (2022) | MRI | 123 | Train/Test | No | No | 2 |
| Acquaviva et al. (2020) | Genomics | 313 | NCV | No | Yes | 3 |
| Ghafouri-Fard et al. (2020) | Genomics | 791 | CV | No | Yes (LIME) | 3 |
| Fuh-Ngwa et al. (2022) | Genomics | 279 | Train/Test | Partial | Yes | 4 |
| Jeyananthan (2024) | Genomics | 78 | CV | No | Yes | 2 |
| Omrani et al. (2024) | Genomics | 225 | Train/Test | No | Limited | 3 |
| Arnal Segura et al. (2025) | Genomics | Large | CV | No | Yes | 3 |
| Montolío et al. (2021) | OCT | 212 | CV | No | Limited | 3 |
| Pérez del Palomar et al. (2019) | OCT | 260 | Train/Test | No | No | 3 |
| He et al. (2019) | OCT | 35 | Train/Test | No | No | 1 |
| Khodabandeh et al. (2024) | OCT | 116 | Train/Test | Partial | Limited | 3 |
| Ciftci Kavaklioglu et al. (2022) | OCT | 699 | CV | No | Limited | 3 |
| Ortiz et al. (2023) | OCT | 148 | Train/Test | No | Limited | 3 |
| Arian et al. (2023) | OCT | 349 | Train/Test | Partial | Limited | 3 |
| Aghababaei et al. (2024) | OCT | 314 | Train/Test | No | Limited | 3 |
| Montolío et al. (2024) | OCT | 102 | Train/Test | No | Limited | 3 |
| Dongil-Moreno et al. (2024) | OCT | 148 | LOOCV | No | Limited | 3 |
| Kenney et al. (2022) | OCT | 2120 | Multi-site | Yes | Limited | 5 |
| Cavaliere et al. (2019) | OCT | 96 | Train/Test | No | No | 2 |
| Yaghoubi et al. (2023) | OCT | 265 | Train/Test | No | Limited | 3 |
| Andorra et al. (2024) | Multimodal | 420 | Multi-site | Yes | Limited | 5 |
| Pinto et al. (2020) | Clinical | 187 | Train/Test | No | No | 2 |
| Pellegrini et al. (2020) | Clinical | 1582 | Multi-trial | Partial | No | 3 |
| Ferrè et al. (2023) | Multimodal | 381 | Train/Test | Partial | Limited | 3 |
